# Traditional Chinese medicine Youguiyin decoction ameliorate glucocorticoid-induced osteonecrosis in rat by modulating ROS/PHD2/HIF-1α oxidative stress signaling pathway in bone marrow mesenchymal stem cells

**DOI:** 10.1186/s13020-025-01113-1

**Published:** 2025-05-03

**Authors:** Hongzhong Xi, Hao Chen, Jiahao Fu, Shuai He, Xin Liu, Guangquan Sun, Bin Du

**Affiliations:** The Affiliated Hospital of Nanjing University of Chinese Medicine, No. 155, Qinhuai Street, Hanzhong Road, Nanjing, 210029 China

**Keywords:** Youguiyin (YGY) decoction, Glucocorticoid-induced osteonecrosis, Oxidative stress, ROS/PHD2/HIF-1α pathway

## Abstract

**Background:**

The incidence of osteonecrosis is increasing annually due to the widespread use of glucocorticoids. Recent evidence suggests a significant association between glucocorticoid-induced osteonecrosis and oxidative stress. Youguiyin (YGY) decoction, a classic formula of traditional Chinese medicine, has been widely used for the prevention of glucocorticoid-induced osteonecrosis. However, its underlying pharmacological mechanisms are still not fully understood.

**Methods:**

UPLC-Q-TOF–MS and network pharmacology were used to elucidate the material basis of YGY decoction and its mechanism for the treatment of glucocorticoid-induced osteonecrosis. The anti-oxidative stress and bone-enhancing effects in vivo were detected by hematoxylin–eosin (HE) staining, serum metabolomics, enzyme-linked immunosorbent assay (ELISA), immunohistochemistry (IHC), and Western Blot (WB). Rat bone marrow mesenchymal stem cells (BMSCs) were induced with dexamethasone (DXMS) for 24 h, followed by YGY medicated serum for 24 h. Significantly up- and down-regulated genes were detected by RNA sequencing. Oxidative stress levels were detected by ROS fluorescence. Alizarin red S staining was used to detect osteogenic effects. WB and ELISA were used to detect the expression of proteins related to the ROS/PHD2/HIF-1a pathway.

**Results:**

The application of YGY decoction significantly promoted bone repair and antagonized excess reactive oxygen species (ROS) generation in glucocorticoid-associated osteonecrosis of the femoral head (GA-ONFH) rats. In addition, YGY medicated serum antagonized DXMS-induced ROS production and promoted osteogenic differentiation in BMSCs. We also found that YGY medicated serum attenuated excess ROS generation while PHD2 expression was significantly increased, HIF-1α expression was significantly decreased and RUNX2 expression was significantly increased.

**Conclusion:**

These results provide compelling in vivo and in vitro evidence that YGY decoction may play a role in promoting glucocorticoid-induced osteonecrosis bone repair by targeting the mediation of the ROS/PHD2/HIF-1α oxidative stress signaling pathway, thus providing a new theoretical basis for the clinical application of YGY decoction to glucocorticoid-induced osteonecrosis.

**Graphical Abstract:**

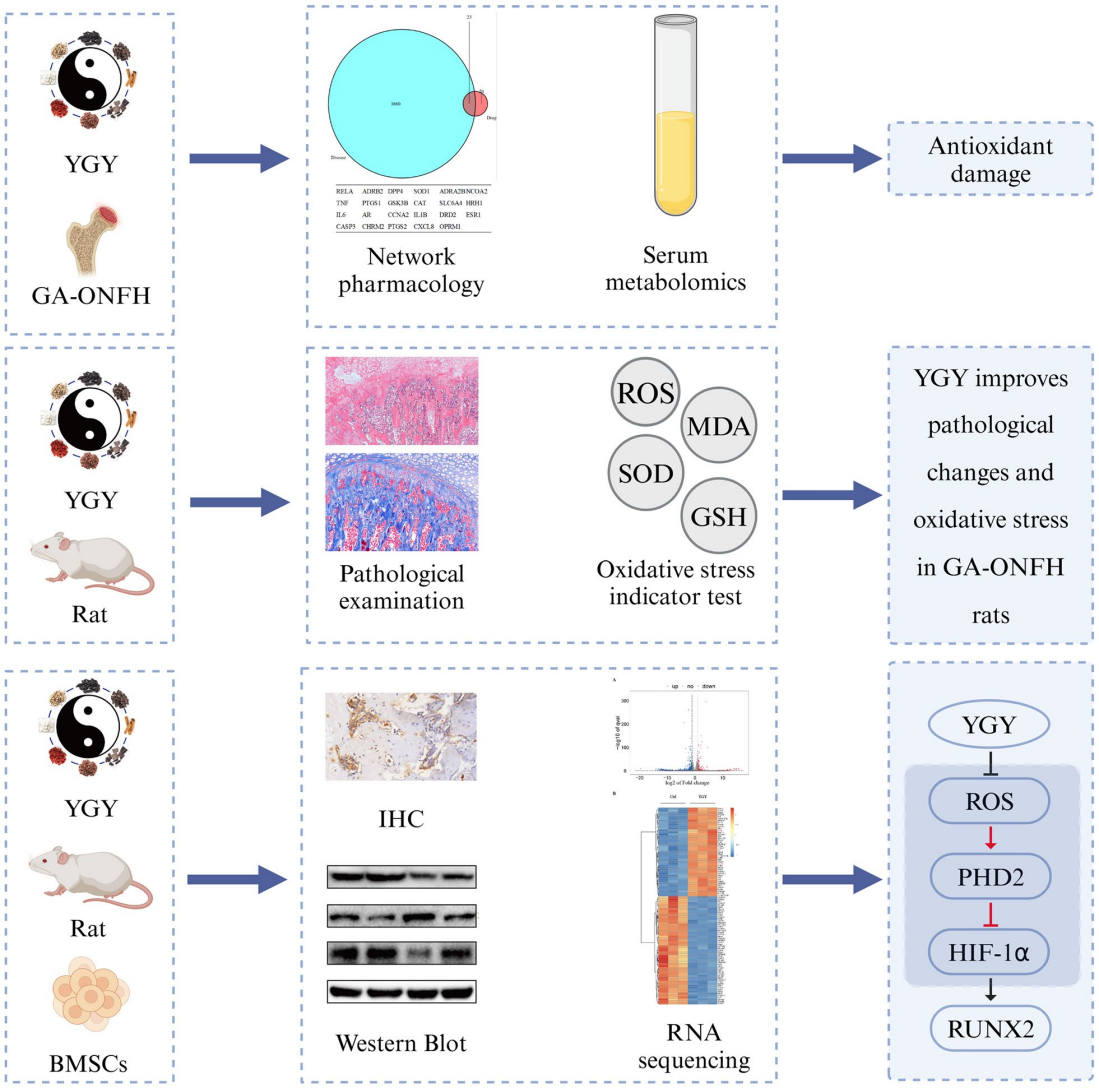

**Supplementary Information:**

The online version contains supplementary material available at 10.1186/s13020-025-01113-1.

## Background

Glucocorticoid administration is a definite risk factor for osteonecrosis. Taking osteonecrosis of the femoral head (ONFH) as an example, glucocorticoid drugs account for 51% of all cases of non-traumatic ONFH [1]. It has been reported that for systemic lupus erythematosus (SLE) patients receiving glucocorticoid therapy, every increase of 10 mg/d in glucocorticoid dosage increases the risk of osteonecrosis by 3.6% [2]. Similarly, patients treated with glucocorticoids for acute respiratory distress have also been observed to develop osteonecrosis during follow-up [3]. However, the pathological and physiological mechanisms underlying glucocorticoid-induced osteonecrosis are not fully understood. Increasing evidence suggests that oxidative stress can cause damage to osteoblast cell lines, thereby playing a profound role in the development of osteonecrosis [4–7]. The essence of oxidative stress is the elevation of reactive oxygen species (ROS) levels within the organism. Excessive ROS disrupts the balance of intracellular redox, leading to irreversible damage through mitochondrial DNA mutation and ultimately resulting in cell apoptosis [8]. Under the context of glucocorticoid-induced osteonecrosis, clearing the excess ROS induced by glucocorticoids can delay the progression of osteonecrosis and serve as an effective approach for preventing and treating glucocorticoid-induced osteonecrosis [9, 10].

It has been established that glucocorticoids induce oxidative stress, leading to the generation of excessive ROS and causing cells to grow in a relatively hypoxic microenvironment [11]. Hypoxia inducible factor-1α (HIF-1α) is a key transcription factor that mediates cellular responses to hypoxia. Under normoxic conditions, proline residues in HIF-1α are hydroxylated by prolyl hydroxylases (PHDs), which are sensitive to oxygen concentration. Subsequently, hydroxylated HIF-1α is ubiquitinated by E3 ubiquitin ligase containing von-Hippel–Lindau tumor suppressor protein (p-VHL) and degraded by proteasomes. However, in the hypoxic environment associated with glucocorticoid-induced osteonecrosis, PHDs are inhibited due to decreased oxygen concentration, impeding the ubiquitination of HIF-1α and resulting in its accumulation in the cell nucleus [12]. Through binding to hypoxia response elements in the nucleus, HIF-1α participates in the regulation of hundreds of gene transcription processes, including angiogenesis, cell proliferation and differentiation, as well as cell apoptosis and autophagy [13]. Previous studies have indicated that among the subunits of PHDs, PHD2 plays a crucial role in regulating HIF-1α homeostasis [14]. Therefore, based on this scientific background, we speculate that the oxidative damage associated with glucocorticoid-induced osteonecrosis may be related to the ROS/PHD2/HIF-1α signaling pathway, and intervention in this signaling pathway may help to delay the progression of osteonecrosis.

Traditional Chinese medicine (TCM) has been used as an alternative therapy for many diseases due to its advantages of minimal side effects. Various single herbs or herbal formulations in TCM can alleviate the main pathological changes of ONFH by regulating bone metabolism, lipid metabolism, and oxidative stress [15–17]. Youguiyin (YGY) decoction is a TCM formulation composed of eight medicinal herbs, which has been used in the treatment of osteonecrosis for over four centuries [18]. In recent clinical applications, YGY decoction has demonstrated excellent preventive effects against glucocorticoid-associated ONFH (GA-ONFH) [19]. Gene expression profiling analysis has shown that the mechanism of YGY decoction in treating GA-ONFH in rats may be related to antioxidant damage [20]. However, it is unclear whether YGY decoction exerts protective effects against oxidative damage in glucocorticoid-induced osteonecrosis through the ROS/PHD2/HIF-1α signaling pathway. In this study, we provide evidence from both in vivo and in vitro perspectives to support the role of YGY decoction in counteracting glucocorticoid-induced osteonecrosis oxidative damage through the ROS/PHD2/HIF-1α signaling pathway, aiming to provide more experimental evidence for the clinical application of YGY decoction.

## Materials

### Preparation of YGY decoction

The YGY decoction consisted of the following ingredients was purchased from the Traditional Chinese Medicine Pharmacy of Jiangsu Provincial Hospital of Traditional Chinese Medicine: 30 g of Shudi (*Rehmannia glutinosa*), 6 g of Shanyao (*Rhizoma dioscoreae*), 3 g of Shanzhuyu (*Fructus corni*), 6 g of Gouqi (*Fructus lycii*), 6 g of Gancao (*Radix glycyrrhizae*), 6 g of Duzhong (*Cortex eucommiae*), 6 g of Rougui (*Cortex cinnamomi*), and 3 g of Fuzi (Monkshood root). The plant names have been checked with http://www.theplantlist.org. The origin, medicinal composites, and processing technology of YGY decoction were standardized based on marker compounds to achieve quality control according to the Chinese Pharmacopoeia 2015 (Chinese Pharmacopeia Commission: Pharmacopoeia of the People’s Republic of China. Chinese Medical Science and Technology Press; Beijing, China, 2010), the same as previously described and published [21]. Briefly, all the herbs were soaked in deionized water for 30 min first, and then boiled for 20 min. The decoction were collected, filtered (filter 0.22 µm), and concentrated into a crude drug with a concentration of 0.5 g/mL. Finally, the decoction were stored at 4℃ for further use.

### Quality control for YGY decoction

To ensure the quality of YGY decoction, ultra-performance liquid chromatography quadrupole time-of-flight mass spectrometry (UPLC-Q-TOF–MS) was employed for the quality control. Briefly, 10 mL YGY decoction extract was added with methanol to a total volume of 25 mL. The mixture was sonicated at 250 W and 40 kHz for 30 min and then was settled at room temperature overnight. Methanol was added to regain a volume of 25 mL solution. After filtrated through a 0.45 μm filter, the solution was used as sample solution for UPLC-Q-TOF–MS experiment. Chromatographic conditions: HSS T3 XP column (2.1 mm × 100 mm, 1.7 μm); flow rate 0.3 mL min⁻^1^; injection volume 2 μL; column temperature 40 ℃; mobile phase consisting of 0.05% formic acid in water (A) and acetonitrile (B) with gradient elution as follows: 0–1.0 min, 100%–98% B; 1.0–2.0 min, 98%–90% B; 2.0–5.0 min, 90%–82% B; 5.0–8.0 min, 82%–80% B; 8.0–10.0 min, 80%–70% B; 10.0–15.0 min, 70%–65% B; 15.0–18.0 min, 65%–90% B; 18.0–20.0 min, 90%–99% B. Mass spectrometric conditions: Electrospray ionization (ESI) source with positive/negative switching mode (0.2 s per scan). Positive ion mode: capillary voltage 3.0 kV, cone voltage 40 V; negative ion mode: capillary voltage − 2.0 kV, cone voltage − 35 V. Collision energy: 20–40 eV for positive mode and 25–60 eV for negative mode (stepped energy). Mass range: m/z 50–1500; acquisition time 25 min; ion source temperature 500 ℃; declustering voltage 90 V; collision gas flow rate 800 L/h. Principal component analysis (PCA) was performed on the UPLC-Q-TOF–MS data using Simic-p.

### Network pharmacology analysis

Genes associated with GA-ONFH were retrieved from the UniProt, OMIM, and GeneCards databases. Overlapping entries from these three databases were merged to eliminate redundancy, yielding a consolidated set of GA-ONFH-related target genes. The bioactive components identified in YGY decoction and the GA-ONFH-related targets were imported into Cytoscape 3.6.1 to visualize biomolecular interactions as a network graph. Using the CytoNCA plugin for network topology analysis, nodes were screened based on centrality metrics: Degree Centrality (DC), Closeness Centrality (CC), Betweenness Centrality (BC), Network Centrality (NC)/Eigenvector Centrality (EC), and Local Average Connectivity (LAC). Nodes with DC values exceeding twice the median value of all nodes were designated as “Big Hubs”, while those surpassing the median value across other metrics were identified as key targets. Finally, Gene Ontology-Biological Process (GO-BP) enrichment analysis of potential targets was performed using the DAVID (Database for Annotation, Visualization and Integrated Discovery) bioinformatics annotation platform.

### In vivo experiments

#### Animal grouping and modeling

This study was approved by the Animal Ethics Committee of Nanjing University of Chinese Medicine (approval number: 2021 DW-12-02). Sample size was determined through review of comparable studies in the literature and confirmed by post-hoc power analysis using experimentally derived effect sizes, ensuring > 90% power to detect significant differences at α = 0.05 [22]. In addition, the sample size of previous studies of the same type was also taken into account, and it was finally decided that the sample size of each group of rats would be 8 rats [23, 24]. Fifty-six specific pathogen-free SD rats (8 weeks old, weighing 240 ± 20 g) were purchased from the Sipeifu Biotechnology Co., Ltd (Beijing, China, No.SCXK 2019–0010). These rats were housed at the Experimental Animal Center of Affiliated Hospital of Nanjing University of Chinese Medicine and had free access to food and water. All rats were kept in cages with four rats per cage and subjected to a 12-h light/dark cycle (22 ℃, 55 ± 5% relative humidity). Prior to the experiment, all animals underwent a one-week adaptation period. After a week of adaptive feeding, forty rats were randomly divided into five groups (n = 8 per group): the control group (Con), the GA-ONFH model group (Mod), the low-dosage YGY decoction group (YGYL, 3 g/kg/d), the high-dosage YGY decoction group (YGYH, 9 g/kg/d) and the alendronate group (ALN, 0.2 mg/kg/d). The GA-ONFH model in rats was established using a combination of lipopolysaccharide (LPS) and methylprednisolone (MPS) [25–27]. In brief, the rats in the Mod group, YGYL group, YGYH group and the ALN group were initially administered LPS (Biosharp, Beijing, China) at a dose of 40 μg/kg via tail vein injection, once every 24 h for a total of three times. Subsequently, they received an intramuscular injection of 60 mg/kg MPS (Shyndec, Shanghai, China) solution in the gluteal region, once every 24 h for a total of ten times. The control group rats were injected with an equivalent volume of normal saline as a control.

#### Drug administration

After the modeling, the rats in the YGYL group and the YGYH group were orally administered at doses of 3 g/kg/d and 9 g/kg/d respectively of YGY decoction for a duration of six weeks. At the same time, the rats in the ALN group were orally administered with alendronate sodium (APExBIO, Houston, USA) at a dose of 0.2 mg/kg/d, for six weeks [28]. The specific dosage for oral administration in rats was calculated based on the dose conversion formula: D_R_ (dose per kg body weight) = D_H_ × R × W_H_/W_R_. In the formula, D_R_ represents the dosage for rats and D_H_ represents the dosage for humans, W_H_ and W_R_ represent the weight of humans and rats, respectively. R is the equivalent dose ratio adjusted by body surface area between humans and rats. The rats in the Con group and Mod group were orally administered with an equivalent volume of 0.9% saline solution as a control.

#### Sample collection

After a 12-h fasting period following the last gavage, each rat was sacrificed. Blood samples were collected via the abdominal aorta, and bilateral femurs were obtained. The blood samples were centrifuged at 12,000 rpm and 4 ℃ for 10 min to obtain the serum, which was then stored in liquid nitrogen. The femoral heads were rapidly isolated and kept in liquid nitrogen for subsequent reagent kit testing and Western Blot analysis.

#### In vivo safety analysis

Body weights of rats in each group were measured and recorded at the start of the experiment and every two weeks thereafter to plot growth curves. Serum levels of the inflammatory cytokines IL-1β and TNF-α were detected using enzyme-linked immunosorbent assay (ELISA) in all experimental groups. Blood routine, liver and kidney functions were analyzed, which included the results of white blood cell count (WBC), neutrophiles percentage (NEU%), alanine aminotransferase (ALT), aspartate aminotransferase (AST), blood urea nitrogen (BUN) and creatinine (Cr).

#### Serum metabolomics analysis

Serum samples were homogenized in liquid nitrogen, extracted with pre-cooled methanol/acetonitrile/water (4:4:2, v/v), and incubated at – 20 ℃ for 1 h. After centrifugation (14,000*g*, 4 ℃, 20 min), the supernatant was dried, reconstituted in acetonitrile/water (1:1, v/v), and centrifuged again (14,000*g*, 4 ℃, 15 min). A pooled quality control (QC) sample (10 μL from each group) was prepared for system validation and stability assessment. Chromatographic separation used an ACQUITY UPLC BEH C18 column (40 ℃, 0.3 mL/min) with mobile phases A (0.1% formic acid/water) and B (acetonitrile) under gradient elution: 5% B (0–1 min), 5–100% B (1–9 min), 100% B (9–12 min), and 5% B (12–15 min). A 5 μL sample was injected for UHPLC-Q-Exactive Orbitrap MS analysis (ESI ± mode). ESI parameters: Gas1/Gas2 = 60, CUR = 30, 320 ℃. Raw data were processed via Compound Discoverer 3.1, followed by multivariate analysis (PCA/OPLS-DA, SIMCA-P 14.1). Metabolites with p < 0.05 and VIP > 1 were selected as potential biomarkers.

#### Oxidative stress and molecular targets analysis

The levels of malondialdehyde (MDA), superoxide dismutase (SOD), and glutathione (GSH) in the serum and femoral heads were measured using assay kits. The levels of PHD2, HIF-1α, and runt-related transcription factor 2 (Runx2) in the serum and femoral heads were measured using ELISA kits.

#### Histological analyses

Femoral head tissues were evaluated via HE and Masson staining to assess GA-ONFH and therapeutic efficacy of YGY decoction [29, 30]. HE-stained sections were analyzed for empty lacunae rate (empty/total lacunae × 100%) and fat follicle area ratio (fat area/marrow cavity area × 100%) at 400 × magnification. Masson staining quantified collagen volume fraction (CVF, blue area/total tissue area × 100%). Immunohistochemistry (IHC) for PHD2, HIF-1α, and Runx2 included dewaxing, peroxidase inactivation, primary/secondary antibody incubation, and microscopic imaging. Staining intensity was scored (0: negative; 2: positive; 3: high positive) using ImageJ’s IHC Profiler, with positivity calculated as: (High% × 3) + (Positive% × 2) + (Low% × 1) [31]. For each group, three randomly selected images were analyzed with three randomly chosen fields of view per image (n = 9 technical replicates).

### In vitro experiments

#### Preparation of YGY medicated serum

Sixteen eight-week-old pathogen-free SD rats (weighing 240 ± 20 g) were randomly divided into the YGY decoction group (n = 8) and the control group (n = 8). The rats in the YGY decoction group received daily oral administration of YGY decoction (9 g/kg) for seven consecutive days. The control group received an equivalent volume of 0.9% saline solution orally. The abdominal aortic blood samples were collected from both groups of rats, and after centrifugation at 4 ℃ and 5000 rpm/min for 10 min, the sera were separated. The obtained sera from both groups were heat-inactivated at 56 ℃ for 30 min and then filtered through a 0.22 μm microporous membrane for sterilization. The YGY medicated serum and blank serum were stored at – 80 ℃ for further use.

#### Cell culture

Rat bone marrow mesenchymal stem cells (BMSCs) were obtained from Procell Life Science and Technology Co., Ltd (Wuhan, China) and maintained in α-MEM culture medium at a 5% CO_2_ concentration and 37 ℃. The culture medium was supplemented with 10% fetal bovine serum (Tianhang, Hangzhou, China) and a mixture of 100 U/mL penicillin and 100 µg/mL streptomycin (Thermo Fisher, USA).

#### Gene knockdown

Lentiviral vector pLKO.1 (HANBIO Biotechnology, INC.) encoding shRNA targeting PHD2, HIF-1α or a scrambled non-targeting control (NC) were transfected into 293 T cells using LipoFiter 3.0 (HANBIO, Shanghai, China). At 48 h post-transfection, virus-containing supernatants were incubated with BMSCs. The sequences of the shRNA targets were as follows: sh-PHD2: 5′-GAGTCAGACCAAGTGCATCAA-3′; sh-HIF-1α:5′-GATCCGCTCACCATCAGTTAC-3′; sh-NC: 5′-GACGAGCACGUGGAGCGCUUUG-3′.

#### Cell model establishment and drug treatment

After treating BMSCs with different concentrations of DXMS for 24 or 48 h, cell proliferation was assessed using the CCK-8 assay. Simultaneously, PCR was performed to detect the expression levels of apoptosis-related genes Bcl-2 and Caspase-3. BMSCs induced by DXMS were then treated with varying concentrations of YGY medicated serum, and apoptosis was evaluated via CCK-8 and PCR. The optimal concentrations of DXMS and YGY medicated serum were determined for subsequent experiments. The cell procedure involved DXMS intervention followed by 24-h treatment with YGY medicated serum. Alternatively, DXMS was applied to PHD2- or HIF-1α-knockdown BMSCs, and YGY medicated serum was subsequently administered for 24 h.

#### Alizarin Red S (ARS) staining

Physiological mineralization is a complex process critical to the development of well-organized structures in bones and teeth [32]. To assess the osteogenic differentiation capacity of cells in each group, we performed an ARS staining assay to evaluate their mineralization levels. Aspirate the culture medium from each group, wash the cells with PBS, and fix them with 4% paraformaldehyde for 10–15 min. After washing again with PBS, stain the cells with 2% ARS solution for 20–30 min. The more pronounced the osteogenic differentiation of BMSCs, the greater the calcium deposits revealed by ARS staining. Observe calcium deposition using an inverted phase-contrast microscope and document with photographs. Calcified nodules were quantified by analyzing three randomly selected fields of view from stained cell images per group, with the experiment independently repeated in triplicate (n = 9 technical replicates per group).

### Molecular mechanisms

#### RNA sequencing and qPCR

BMSCs at 80–90% confluency were treated with 10% YGY medicated serum for 24 h. Total RNA was extracted using TRIzol, quantified (NanoDrop ND-1000), and assessed for integrity (RIN > 7.0, Bioanalyzer 2100/agarose gel). Poly(A) RNA was enriched via two rounds of Oligo(dT) bead purification, fragmented (94 ℃, 5–7 min), and reverse-transcribed into cDNA. Double-stranded cDNA libraries were constructed using SuperScript II, E. coli DNA polymerase I, and dUTP, then sequenced on Illumina NovaSeq 6000. Raw reads were processed (fastp), aligned to the rat genome (HISAT2), and assembled (StringTie). Transcript expression was quantified as FPKM, and differential mRNA analysis (|fold change|> 2, p < 0.05 via edgeR) was performed. Results were validated across three independent biological replicates (n = 3), with statistical analysis performed across replicates to ensure robustness. Total RNA was extracted from cells and reverse transcribed into cDNA for real-time qPCR analysis. The primers for the target gene were synthesized by Wuhan Servicebio Technology Co., Ltd, and all sequences are shown in Table 1. Gene expression was normalized using the 2^−ΔΔCт^ method. The PCR assay was performed in six independent experimental replicates (n = 6).

**Table 1:** Primer sequences for RT-qPCR

Primer name	Forward primer (5′-3′)	Reverse primer (5′-3′)
GCLC	GGGGTGACGAGGTGGAGTA	GTGGTTGGGGTTTGTCCTCT
SOD2	ATTGCCGCCTGCTCTAATCA	AGTGCTGCAATGCTCTACAC
PHD2	CCTGCATACGCCACAAGGTA	CCTCACACCTTTCTCACCTGT
HIF-1α	GGCGAGAACGAGAAGAAAAATAGG	AGATGGGAGCTCACGTTGTG
RUNX2	CACAAGTGCGGTGCAAACTT	TGAAACTCTTGCCTCGTCCG
β-actin	AGCCTTCCTTCCTGGGTATGG	AAGGGTGTAAAACGCAGCTC

#### ROS detection

The cell culture medium of each group was aspirated and washed twice with PBS. The washed PBS was aspirated and the corresponding volume of DCFH-DA working solution was added, and the cells were incubated at 37 ℃ and 5% CO_2_ in an incubator protected from light for 30 min. After two washes with PBS, the cells were observed by fluorescence microscope.

After digestion of BMSCs with trypsin, the cells were collected by centrifugation and washed three times with PBS at 4 ℃. An appropriate amount of PBS was used to resuspend the cells, which were then sonicated to disrupt the cell suspension. The resulting fragmented cells were frozen below – 20 ℃, thawed at room temperature, and subjected to three cycles of freezing and thawing to ensure complete cell swelling and rupture. Subsequently, the samples were centrifuged at 1500*g* for 10 min at 4 ℃, and the supernatant was collected for the detection of ROS, MDA, SOD, and GSH levels using an ELISA assay kit.

#### Western blot

The protein expression levels of PHD2, HIF-1α, and Runx2 were detected using western blot. Proteins (20 μg per lane) were separated on a 10% SDS-PAGE gel and transferred onto a PVDF membrane. After incubation with 5% skim milk for 1 h, the membrane was incubated with primary antibodies (anti-PHD2, 1:5000; anti-HIF-1α, 1:2000; anti-Runx2, 1:1000; β-actin, 1:10000) at 4 ℃ for 12 h. Subsequently, the membrane was incubated with corresponding secondary antibodies at room temperature for 1 h. Finally, the protein bands were visualized using a Bio-Rad Gel Imaging System (Chemidoc XRS +, USA). Western blot analysis was independently performed in triplicate (n = 3 biological replicates).

### Statistical analysis

All numerical data are presented as mean ± standard deviation (SD). Statistical differences were determined using an unpaired t-test by SPSS 24.0 software. A p-value less than 0.05 was considered statistically significant.

## Results

### Identification of chemical compounds in YGY decoction by UPLC-Q-TOF–MS

**Fig. 1:**
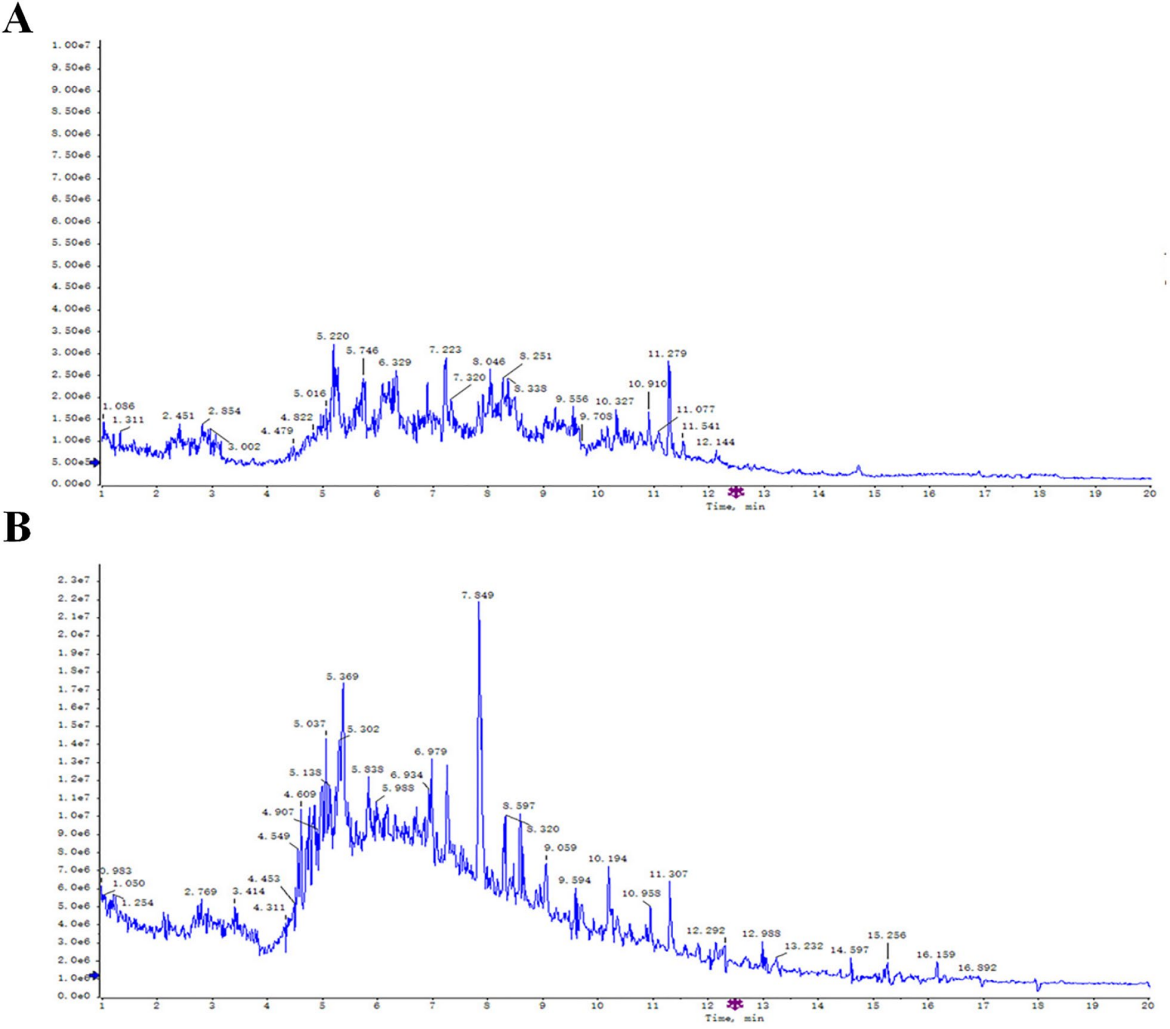
Base peak chromatograms of YGY decoction obtained by UPLC-Q-TOF–MS in both positive (**A**) and negative (**B**) ion mode

In the UPLC-Q-TOF–MS experiment, a total of 76 active components of YGY decoction were detected (Fig. 1). Based on the measured accurate relative molecular weights, combined with the secondary mass spectrometry fragments and the established compound database of individual herbs in YGY decoction from literature reports, qualitative analysis and chemical component identification of the chromatographic peaks were conducted. A total of 33 potential major components of YGY decoction were identified (Table 2). Among all these compounds, 5,7,3′-trimethoxy-levorotatory-epicatechin, delcosine, neo-rehmannioside, echinacoside, and rutin were likely to be the most significant active components of YGY decoction.

**Table 2:** Characterization of the abundance of compounds in YGY decoction by UPLC-Q-TOF–MS

NO.	Name	Formula	Peak	Source
01	5,7,3′-trimethoxy-levorotatory-epicatechin	C_18_H_19_O_6_	142,405.25	SYR, DZ, RG, GC,GQ
02	Delcosine	C_33_H_46_NO_9_	105,419.67	–
03	Neo-rehmannioside	C_21_H_36_O_9_	86,561.50	SDH, DZ, GQ, FZ
04	Echinacoside	C_35_H_45_O_20_	70,346.67	SDH
05	Rutin	C_27_H_30_O_16_	64,394.33	GQ
06	(A-b)-14α-benzoyloxy-N-ethyl-3α,10β,13β,15α-tetrahydroxy-lα,6α,8β,16β,18-pentamethoxyaconitane	C_33_H_47_NO_12_	50,353.00	-
07	Martynoside D	C_31_H_40_O_15_	44,017.00	SDH
08	Martynoside	C_31_H_40_O_15_	40,951.50	SDH
09	Aconine	C_25_H_41_NO_9_	26,258.00	FZ
10	Koaburaside	C_14_H_20_O_9_	21,709.00	GC, GQ
11	Secoisolariciresinol	C_20_H_26_O_6_	19,973.50	RG
12	Deoxyaconitine	C_34_H_47_NO_9_	19,493.13	FZ
13	Methyl eugenate-4-o-β-d-glucoside	C_16_H_23_O_11_	18,348.33	SYR
14	Isoswertisin hexaacetate	C_34_H_34_O_16_	15,931.50	SDH
15	22-Methoxyouabain	C_30_H_46_O_13_	15,894.00	FZ
16	5-hydroxy-2-hydroxymethyl pyridine	C_30_H_48_O_7_	14,904.20	SDH, SYR, DZ, GQ, FZ
17	1-hydroxypinoresinol 4′,4ʺ-di-o-β-d-glucopyranoside	C_32_H_42_O_17_	13,677.25	GC
18	Cinncassiol D1 glucoside	C_26_H_42_O_10_	10,757.33	–
19	Pinoresinol	C_32_H_42_O_16_	10,752.00	DZ
20	Cinnzeylanol	C_20_H_32_O_7_	10,486.25	SYR, SY, GQ
21	Lasiokaurin C	C_20_H_30_O_6_	9636.33	–
22	Hypaconitine	C_33_H_45_NO_10_	8944.50	–
23	Castanoside C	C_30_H_38_O_15_	8904.50	SDH, SY
24	Jionoside D	C_29_H_36_O_13_	8900.50	SDH, SY
25	Presenegenin	C_30_H_46_O_7_	8818.25	RG, DZ, GQ, FZ
26	l-Hyoscyamine	C_17_H_23_NO_3_	8022.33	SYR
27	Lyciumamide B	C_36_H_36_N_2_O_8_	7028.50	SYR
28	Deacetylaconitine	C_32_H_45_NO_10_	6874.50	–
29	Glucosyl-vitexin	C_27_H_30_O_15_	6756.17	–
30	1-acetyl-β-carboline	C_13_H_10_N_2_O	6051.67	SDH, GQ
31	1-hydroxypinoresinol-4′-o-β-d-glucopyranoside	C_26_H_32_O_12_	5471.50	–
32	Atropine	C_17_H_23_NO_3_	5370.50	SYR
33	Secoisolariciresinol	C_32_H_46_O_16_	5008.50	–

### Antioxidant damage may be one of the mechanism by which YGY decoction counteracts glucocorticoid-induced osteonecrosis

**Fig. 2:**
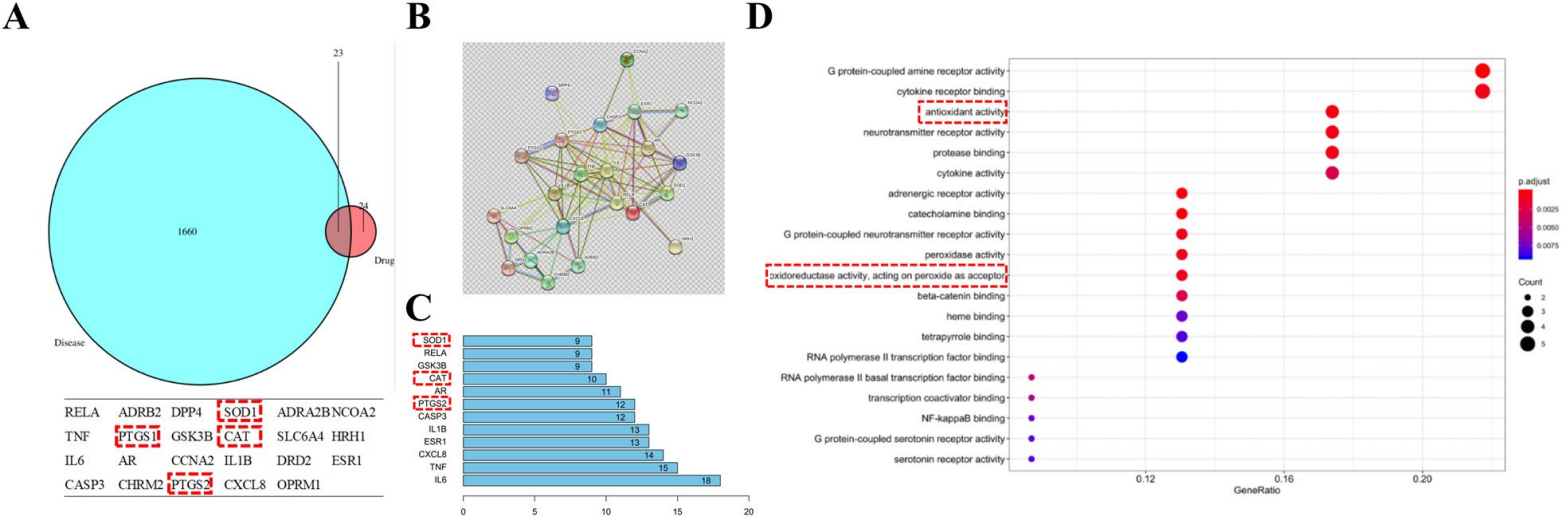
Network pharmacology analysis results of YGY decoction and GA-ONFH. **A** Venn diagram of target genes associated with abundant components of YGY decoction and GA-ONFH. **B** PPI network diagram of potential target proteins for the treatment of GA-ONFH with YGY decoction. **C** Hub proteins and degree values closely associated with YGY treatment of GA-ONFH. **D** The enrichment results of GO-BP for YGY intervention with GA-ONFH

A total of 71 key targets linked to YGY decoction’s components were identified (Table S1), with 23 overlapping genes associated with glucocorticoid-induced osteonecrosis (Fig. 2A). Network analysis of compound-target interactions revealed 34 nodes, with 8 hub targets showing high centrality (Table S2). Protein–protein interaction (PPI) network analysis highlighted critical hub targets (degree > 9) (Fig. 2B, C). GO enrichment suggested YGY decoction’s therapeutic effects involve antioxidant pathways (Fig. 2D). Metabolomic analysis identified 39 differentially expressed proteins in GA-ONFH rats, with 10 significantly modulated by YGY decoction (Table S3). These findings support YGY decoction’s role in alleviating GA-ONFH via anti-oxidative damage.

### YGY decoction promoted femoral bone repair in GA-ONFH rats

**Fig. 3:**
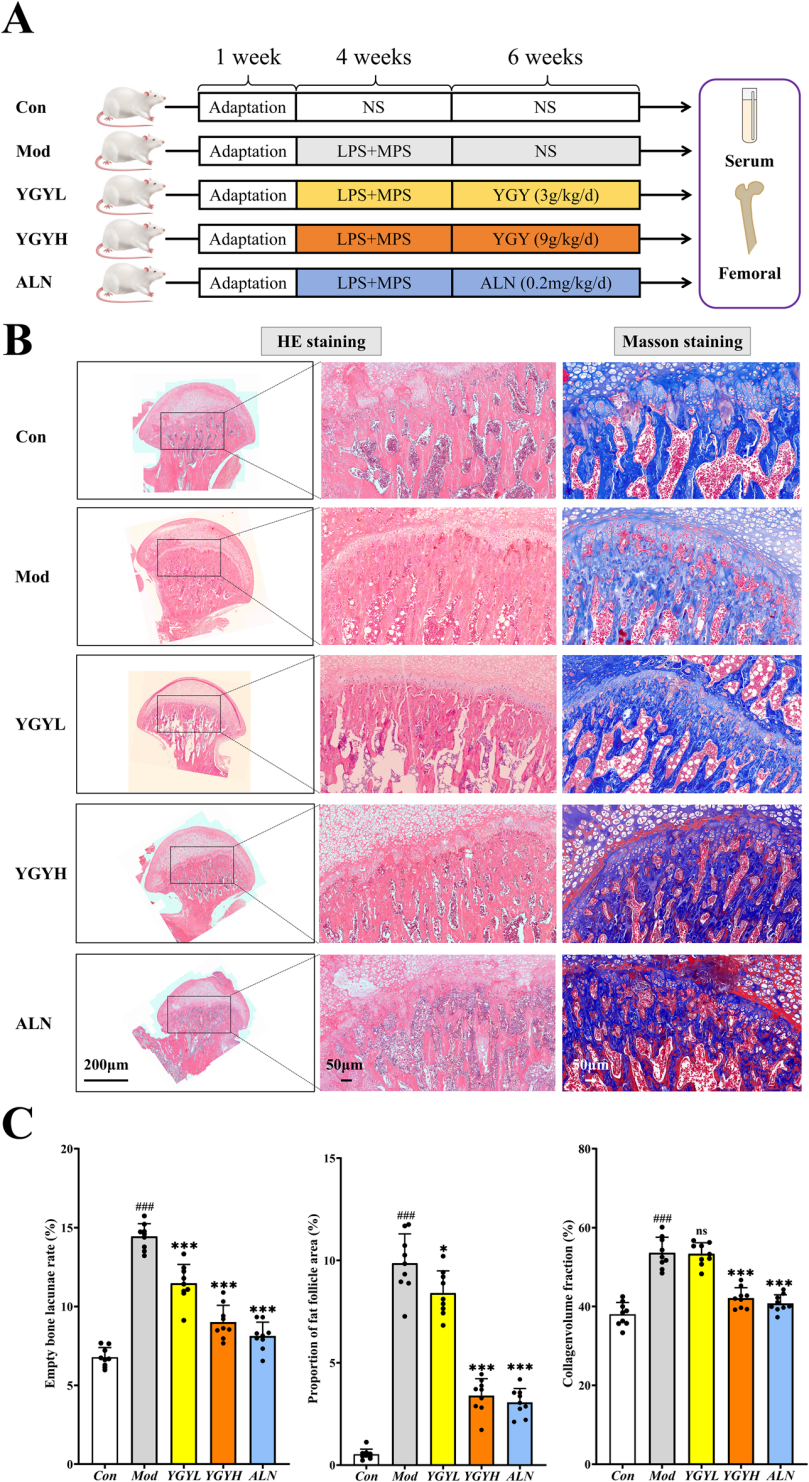
The reparative effect of YGY decoction on the femoral head of GA-ONFH rats. **A** Specific procedure of animal experiment. **B** Representative micrograph of HE and Masson staining of the femoral head. Scale bar: 200/50 μm. **C** Quantitative analysis of the results of HE staining and Masson staining (n = 9 technical replicates). Statistical analysis: Unpaired t-test. ^###^*P* < 0.001 compared vs Con group. ^ns^*P* > 0.05,*P < 0.05 and ***P < 0.001 vs Mod group

Rat body weight gradually decreased during modeling, recovered post-modeling, and showed greater recovery with YGY decoction intervention (Fig. S1 A). The Mod group exhibited significantly elevated inflammatory cytokines, while YGY decoction reduced their levels (Fig. S1B). Routine blood tests and hepatic/renal function parameters (WBC, NEU%, ALT, AST, Cr, BUN) remained normal across groups, indicating no biological toxicity of YGY decoction (Fig. S1 C). In the Mod group 75% of rats developed osteonecrosis, with 6 cases showing significant necrosis. YGY decoction-treated groups (YGYL: 5; YGYH: 7) and ALN group (6) also exhibited necrosis, but no mortality occurred during modeling. Figure 3A illustrates the general flow of the animal experiments. Histological analysis revealed structural disruption in Mod rats, including hardened bone areas, narrowed trabecular spaces, empty lacunae, contracted osteocyte nuclei, and necrotic bone marrow. Mod rats also displayed increased intramedullary adipose tissue, often forming cyst-like structures. In contrast, YGY decoction treatment reduced osteocyte nuclear contraction and fat accumulation, with dose-dependent efficacy (Fig. 3B). Quantitative analysis confirmed elevated empty lacunae rate and fat area in Mod rats, both mitigated by YGY decoction. Similarly, Masson staining showed higher necrotic area (blue) in Mod rats, which decreased post-YGY decoction intervention (Fig. 3C).

### YGY decoction enhanced osteogenesis in GA-ONFH rats via the ROS/PHD2/HIF-1α pathway

**Fig. 4:**
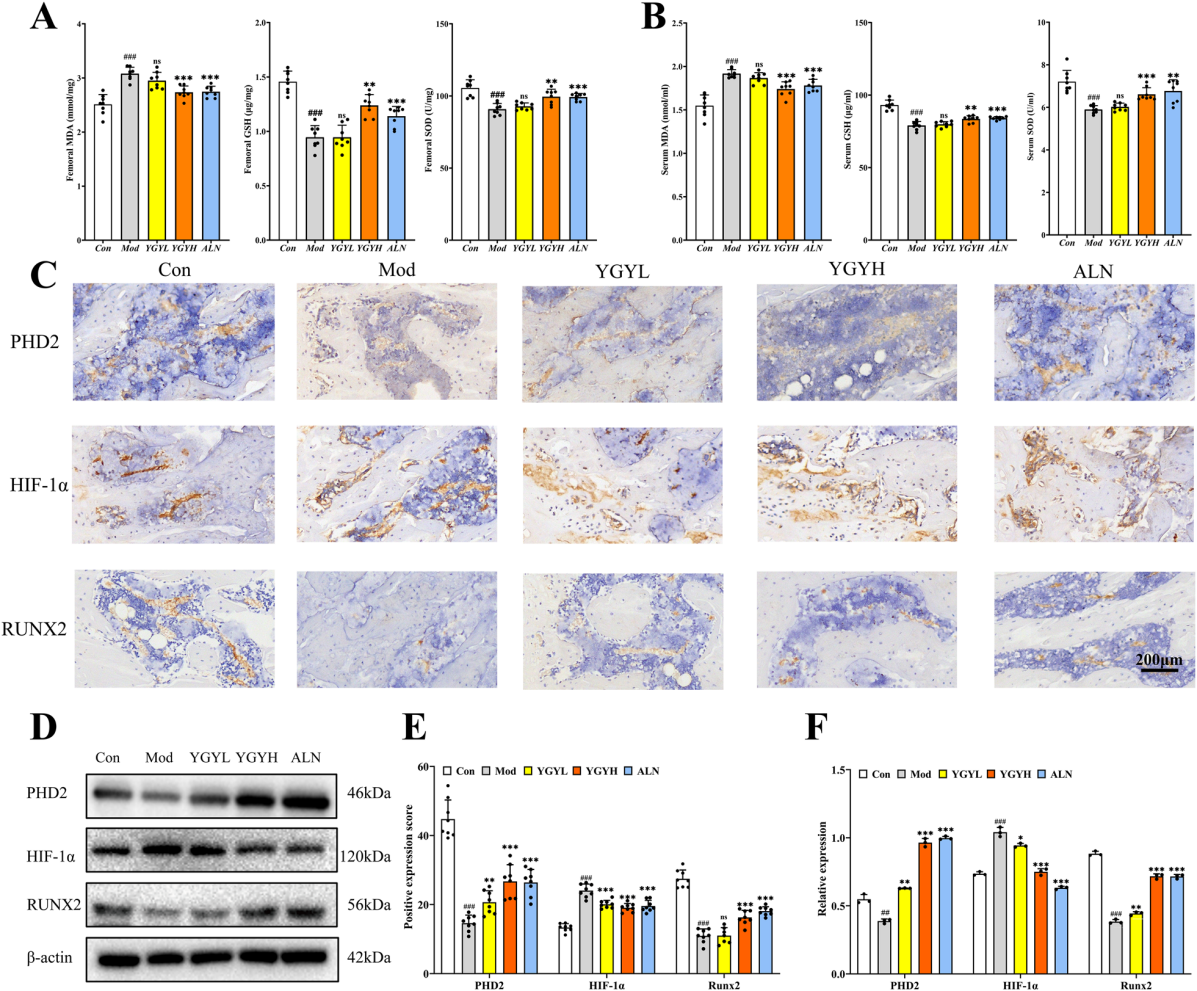
The effects of YGY decoction on ROS, PHD2, HIF-1α, and osteogenic expression in GA-ONFH rats. **A** Oxidative stress levels in the femoral head of rats in all groups. **B** Oxidative stress levels in the serum of rats in all groups. **C** Immunohistochemical staining results of the femoral head in each group of rats. Scale bar: 200 µm. **D** Western blot results of the femoral head in each group of rats. **E** Quantitative analysis of immunohistochemical positive expression in each group of rats (n = 9 technical replicates, 3 fields/sample). **F** Quantitative analysis of expression of western blot in each group of rats (n = 3 independent experiments). Statistical analysis: Unpaired t-test. ^##^*P* < 0.01 and ^###^*P* < 0.001 vs Con group. ^ns^*P* > 0.05,*P < 0.05, **P < 0.01 and ***P < 0.001 vs Mod group

To evaluate ROS levels in rat femoral heads, MDA, GSH, and SOD were measured. In Mod group, MDA increased significantly in serum and femoral tissues but decreased with YGY decoction treatment. Conversely, reduced antioxidant markers (GSH, SOD) in the Mod group were restored by YGY decoction (Fig. 4A, B). Immunohistochemistry showed decreased PHD2 and elevated HIF-1α in the Mod group, which YGY decoction reversed dose-dependently (Fig. 4C, E). ELISA and Western blot analyses confirmed these effects (Fig. S2 A, B; Fig. 4D, F). YGY decoction also partially restored osteogenic marker RUNX2 expression. Collectively, YGY decoction promoted bone repair in GA-ONFH rats by modulating the ROS/PHD2/HIF-1α pathway.

### Results of RNA sequencing (RNA-seq)

**Fig. 5:**
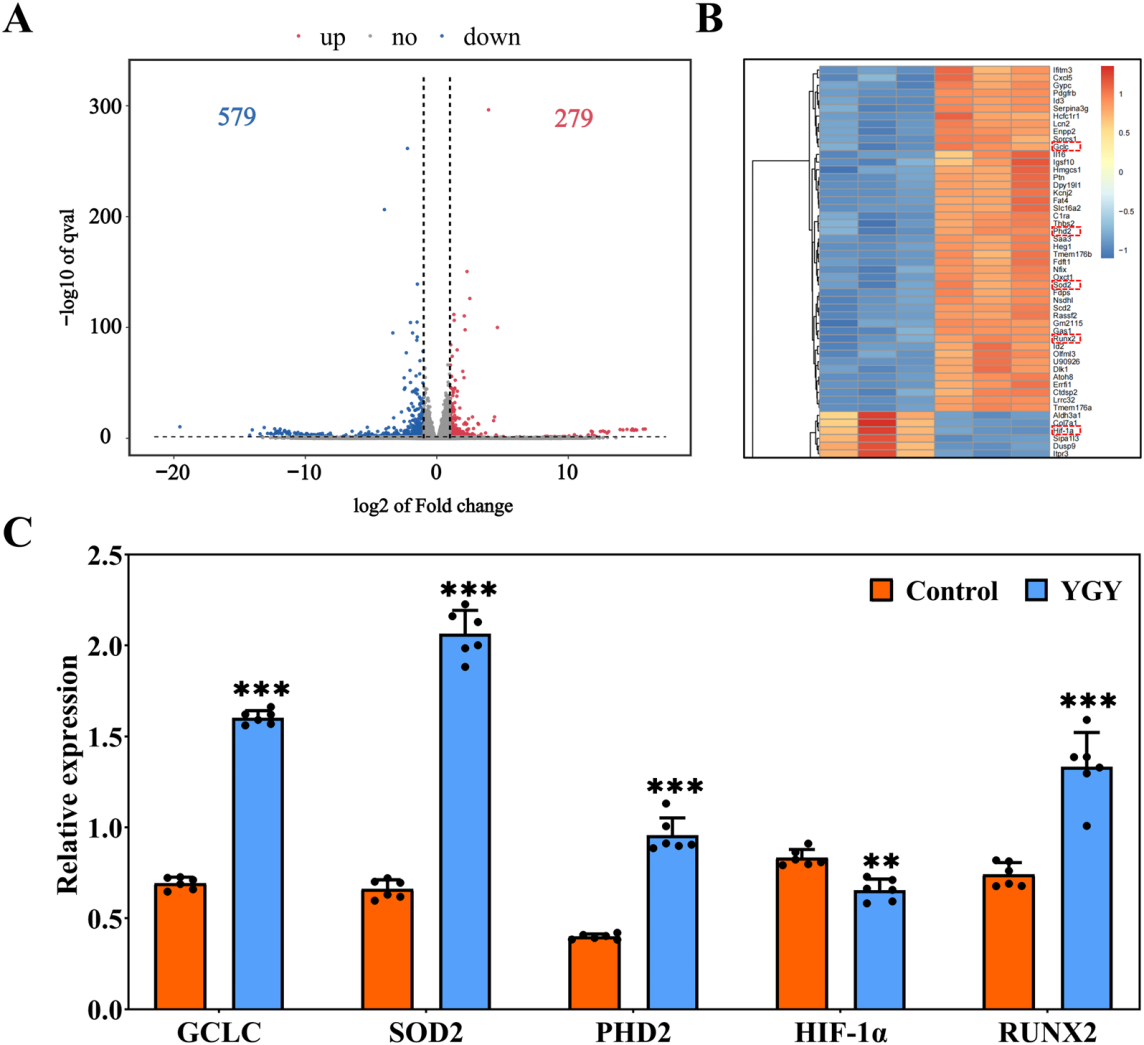
RNA sequencing and qPCR results. **A** The volcano plot of different expressed genes. **B** Hierarchical clustering analysis of different expressed genes. **C** The relative gene expression levels of GCLC, SOD2, PHD2, HIF-1α and RUNX2 (n = 6 independent experiments). Statistical analysis: Unpaired t-test. **P < 0.01 and ***P < 0.001 vs Control group

To further investigate the molecular mechanisms of YGY medicated serum on DXMS-induced BMSCs, we performed RNA-seq analysis of DXMS-induced BMSCs cultured with YGY medicated serum intervention. Figure 5A showed a volcano plot of different expressed genes (DEGs), where gray indicated genes with non-significant differences and red and blue indicated genes with significantly altered expression. Among all DEGs, there were 279 genes with up-regulated expression (red) and 579 genes with down-regulated expression (blue). Based on the top 100 DEGs shown in the heatmap (Fig. 5B), when compared to the control group, significant upregulation was observed in Glutamate-Cysteine Ligase Catalytic (GCLC), PHD2, SOD2 and RUNX2, while significant downregulation was observed in HIF-1α. Subsequently, real-time qPCR analysis was performed to detect and validate all these significant-regulated genes. As can be seen from Fig. 5C, the results of qPCR were consistent with those of RNA-seq.

### YGY medicated serum promoted osteogenic differentiation of DXMS-induced BMSCs through the ROS/PHD2/HIF-1α signaling pathway

**Fig. 6:**
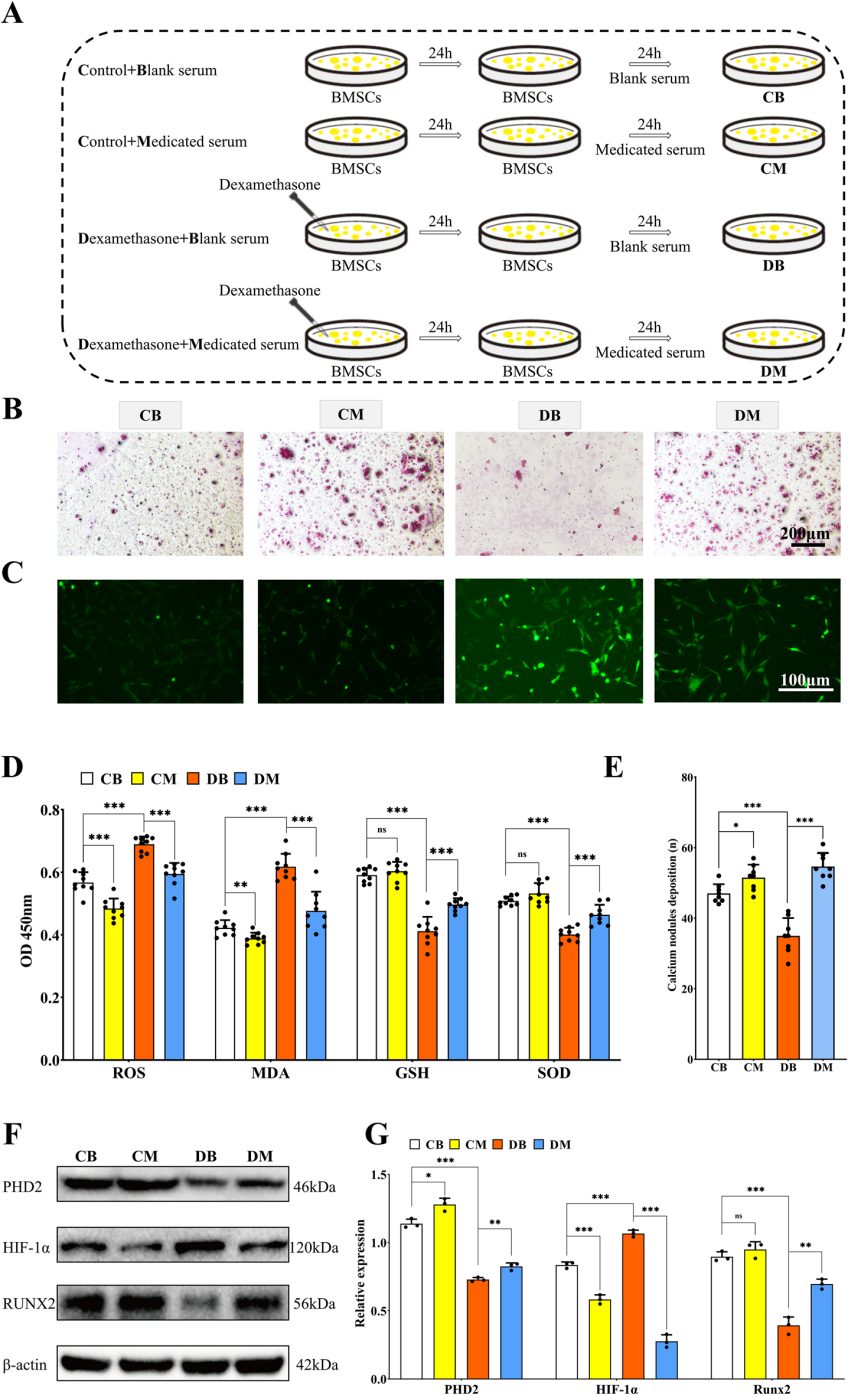
Promoting effect of YGY medicated serum on osteogenic differentiation of dexamethasone-induced BMSCs. **A** Specific procedure and grouping of cell experiments. **B** Alizarin red staining results of each group of cells. Scale bar: 200 μm. **C** ROS fluorescence results for each group of cells. Scale bar: 100 μm. **D** Levels of oxidative stress for each group of cells (n = 6 technical replicates). **E** Number of calcium nodule deposits for each group of cells (n = 9 technical replicates). **F** Western blot results for each group of cells. **G** Quantitative analysis results of Western Blot (n = 3 independent experiments). Statistical analysis: Unpaired t-test. ^ns^P > 0.05,*P < 0.05, **P < 0.01 and ***P < 0.001

It has been shown that DXMS can induce oxidative damage [33–36]. 50 μM DXMS (24 h) induced peak BMSC apoptosis with minimal Bcl2 and maximal Caspase3 expression (Fig. S3 A, B). YGY medicated serum demonstrated concentration-dependent rescue: higher doses elevated proliferation while upregulating Bcl2 and suppressing Caspase3 (Fig. S3 C, D). These findings informed our selection of 50 μM DXMS with 10% YGY serum for subsequent assays. Figure 6A outlined the workflow of this in vitro experiment. As demonstrated in Fig. 6B and E, DXMS treatment for 24 h significantly reduced calcium nodule formation in BMSCs, whereas YGY medicated serum restored calcium nodule deposition, indicating its ability to promote osteogenic differentiation in DXMS-induced BMSCs. Furthermore, PCR analysis of osteogenic genes in each group of cells revealed that the osteogenic expression was significantly decreased after DXMS intervention in BMSCs, while YGY medicated serum significantly rescued the DXMS-induced downregulation of osteogenic genes (Fig. S4 A). Figure 6C revealed that the ROS fluorescence intensity was markedly increased in the DB group, suggesting DXMS significantly elevated intracellular ROS levels. However, YGY medicated serum effectively antagonized this ROS elevation, which was further confirmed by ELISA results showing reduced ROS levels in BMSCs treated by YGY medicated serum (Fig. 6D). Furthermore, WB analysis demonstrated that DXMS downregulated PHD2 expression and upregulated HIF-1α expression in BMSCs, while YGY medicated serum restored PHD2 expression and suppressed HIF-1α levels (Fig. 6F, G). Additionally, the expression of the osteogenic marker RUNX2 was significantly decreased in DXMS-treated BMSCs but restored by YGY medicated serum intervention. These findings collectively suggested that YGY medicated serum promotes osteogenic differentiation in DXMS-induced BMSCs likely through modulating the ROS/PHD2/HIF-1α signaling pathway.

### PHD2 knockdown attenuated the promoting effect of YGY medicated serum on the osteogenic differentiation of DXMS-induced BMSCs

**Fig. 7:**
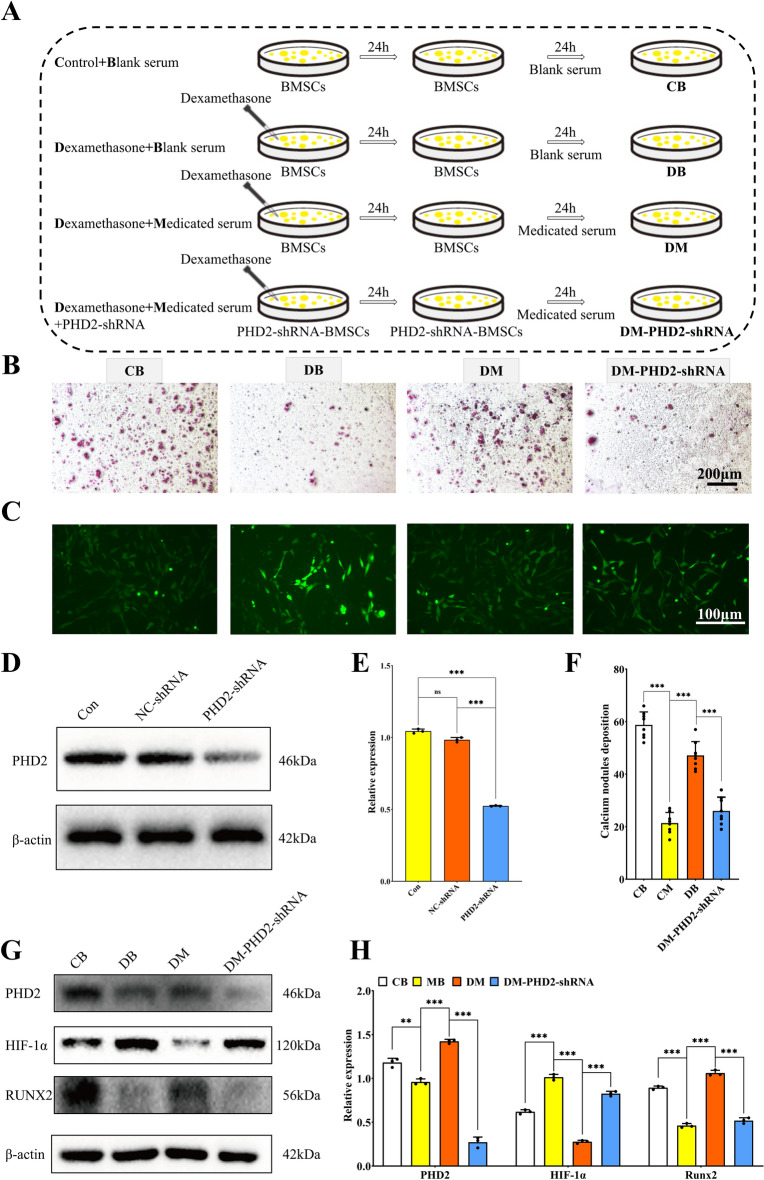
PHD2 knockdown hindered the promotion of osteogenic differentiation of dexamethasone-induced BMSCs by YGY medicated serum. **A** Specific procedure and grouping of cell experiments. **B** Alizarin red staining results for each group of cells. Scale bar: 200 μm. **C** ROS fluorescence results for each group of cells. Scale bar: 100 μm. **D** Western blot analysis of PHD2 knockdown in BMSCs. **E** Quantitative analysis results of Western Blot of PHD2 knockdown in BMSCs (n = 3 independent experiments). **F** Number of calcium nodule deposits for each group of cells (n = 9 technical replicates). **G** Western blot results for each group of cells. **H** Quantitative analysis results of western blot (n = 3 independent experiments). Statistical analysis: Unpaired t-test. ^ns^*P* > 0.05, **P < 0.01 and ***P < 0.001

Figure 7A illustrated the schematic diagram of the experimental workflow in this study. Prior to initiating the experiment, we first performed PHD2 knockdown in BMSCs. As shown in Fig. 7D and E, compared to the Con group, BMSCs transfected with the PHD2-shRNA lentiviral plasmid exhibited significantly reduced PHD2 expression, while those transfected with the empty vector showed no notable changes, confirming successful lentiviral transfection and PHD2 knockdown in BMSCs. Furthermore, after PHD2 knockdown, BMSCs subjected to DXMS induction and YGY medicated serum intervention demonstrated significantly diminished osteogenic differentiation compared to the DM group (Fig. 7B, F). Meanwhile, after knockdown of the PHD2 gene in BMSCs, the expression of osteogenic genes was significantly reduced compared to the DM group (Fig. S4B). This indicated that PHD2 knockdown attenuated the enhancing effect of YGY medicated serum on the osteogenic differentiation of DXMS-induced BMSCs. Results from the ROS fluorescence probe assay revealed that PHD2 knockdown did not alter intracellular ROS levels in YGY medicated serum-treated, DXMS-induced BMSCs (Fig. 7C). Intriguingly, WB analysis showed that PHD2 knockdown markedly upregulated HIF-1α expression while downregulating the osteogenic marker RUNX2 (Fig. 7G, H). These findings suggested that PHD2 knockdown reduced the pro-osteogenic effects of YGY medicated serum in DXMS-induced BMSCs, potentially linked to enhanced HIF-1α expression and suppressed osteogenic activity.

### HIF-1α knockdown enhanced the promoting effect of YGY medicated serum on the osteogenic differentiation of DXMS-induced BMSCs

**Fig. 8:**
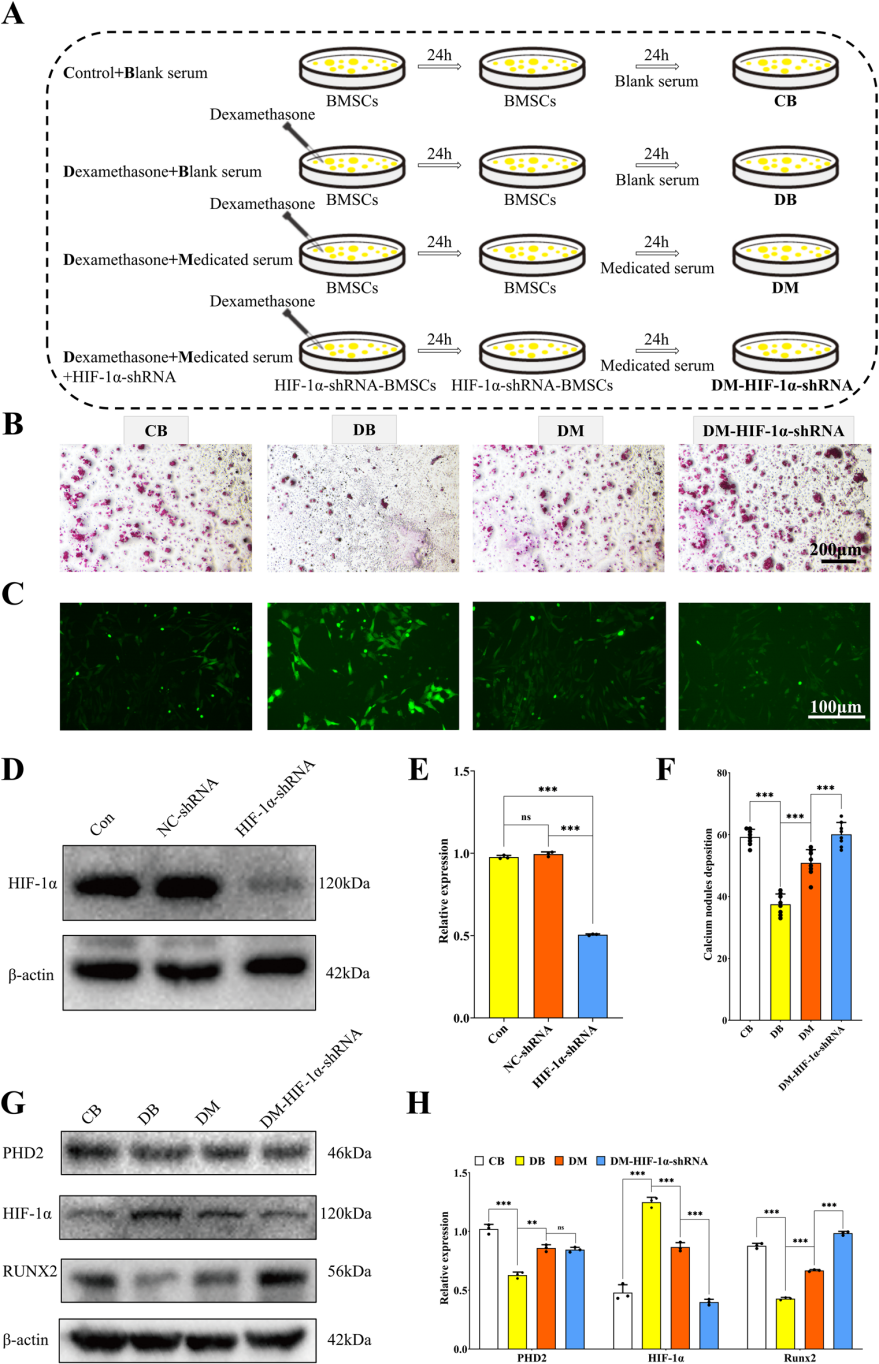
HIF-1α knockdown promoted the promotion of osteogenic differentiation of dexamethasone-induced BMSCs by YGY-medicated serum. **A** Specific procedure and grouping of cell experiments. **B** Alizarin red staining results for each group of cells. Scale bar: 200 μm. **C** ROS fluorescence results for each group of cells. Scale bar: 100 μm. **D** Western blot analysis of HIF-1α knockdown in BMSCs. **E** Quantitative analysis results of Western Blot of HIF-1α knockdown in BMSCs (n = 3 independent experiments). **F** Number of calcium nodule deposits for each group of cells (n = 9 technical replicates). **G** Western blot results for each group of cells. **H** Quantitative analysis results of western blot (n = 3 independent experiments). Statistical analysis: Unpaired t-test. ^ns^*P* > 0.05, *P < 0.05, **P < 0.01 and ***P < 0.001

This experiment was conducted following the general procedure outlined in Fig. 8A. As shown in Fig. 8D and E, HIF-1α-knockdown lentivirus successfully infected BMSCs, with significantly reduced HIF-1α protein expression compared to the Con group. ARS staining demonstrated that HIF-1α knockdown markedly enhanced the promoting effect of YGY medicated serum on DXMS-induced osteogenic differentiation of BMSCs (Fig. 8B, F). Meanwhile, PCR analysis revealed that knockdown of HIF-1α in BMSCs could promote the expression of osteogenic genes (Fig. S4 C). Furthermore, YGY medicated serum reduced DXMS-induced excessive ROS production in BMSCs, while HIF-1α knockdown showed no impact on ROS levels (Fig. 8C). WB analysis revealed no significant change in PHD2 expression after HIF-1α knockdown, but a notable increase in the expression of the osteogenic gene RUNX2 (Fig. 8G, H). These results indicated that HIF-1α knockdown did not affect ROS or PHD2 expression, but rather enhanced the pro-osteogenic effects of YGY medicated serum.

## Discussion

The pathogenesis of glucocorticoid-induced osteonecrosis involves multiple factors, and apoptosis of bone cells induced by oxidative stress is one of the causes [37]. In previous clinical practices, YGY decoction has been proven to have significant efficacy in the prevention and treatment of GA-ONFH. Study has shown that YGY decoction could promote bone formation during GA-ONFH [19]. It has also been shown that that YGY decoction may exert its intervention in glucocorticoid-induced osteonecrosis through antioxidative damage [20]. In the in vivo experiments of this study, we found that the YGY decoction promoted femoral bone repair in GA-ONFH rats. Additionally, in vitro cell experiments demonstrated that the YGY medicated serum enhanced osteogenic differentiation of DXMS-induced BMSCs. These results further confirm the clinical efficacy of the YGY decoction in treating glucocorticoid-induced osteonecrosis.

As evidenced by the identified major active components of YGY decoction, the formulation itself represents a composite of antioxidant compounds, including rehmannioside (attenuates oxidative stress, inflammation, and apoptosis), rutin (a natural ROS scavenger), and martynoside (combats oxidative injury) [38–42]. Network pharmacology predictions link YGY decoction’s anti-GA-ONFH effects to modulation of antioxidant genes such as SOD1, CAT, and PTGS2. Specifically, SOD1 and CAT regulate ROS homeostasis: SOD1 converts superoxide to hydrogen peroxide, which CAT further breaks down into water and oxygen [43, 44]. RNA-seq revealed that YGY medicated serum significantly upregulated SOD2 (mitochondrial SOD) and GCLC (a rate-limiting enzyme in glutathione synthesis) in BMSCs, enhancing ROS clearance through both SOD-mediated catalysis and glutathione-dependent pathways [45]. Concurrently, suppression of PTGS2 (a gene linked to iron-mediated oxidative stress) further mitigates ROS accumulation [46–48]. Together, these mechanisms explain how YGY decoction counteracts glucocorticoid-induced ROS overload in BMSCs, thereby preserving osteogenic differentiation and delaying osteonecrosis progression.

Oxidative stress is one of the critical factors contributing to osteocyte apoptosis [4, 49]. Under physiological conditions, ROS participate in the regulation of intracellular and extracellular redox balance, playing a vital role in maintaining cellular homeostasis [50]. However, under the influence of glucocorticoids, excessive ROS production disrupts intracellular redox equilibrium. This oxidative overload compromises cellular integrity, impairing intercellular material exchange and signal transduction [51]. Our findings align with the observation that glucocorticoids induce oxidative stress, triggering excessive ROS generation in rats, thereby exacerbating osteocyte dysfunction.

In this study, compared to the untreated Con group, glucocorticoid intervention significantly elevated ROS levels in both systemic serum and local femoral head tissue. Notably, administration of the YGY decoction markedly reduced ROS abundance, demonstrating that clearing excess ROS may be a key mechanism by which YGY alleviates GA-ONFH in rats. Consistent with these in vivo findings, in vitro cell experiments revealed that glucocorticoid exposure triggered rapid ROS accumulation in BMSCs, whereas intervention with YGY medicated serum effectively cleared the excessive ROS. Some evidence suggests that elevated levels of ROS can lead to inactivation of PHD2 [52–55]. This may be due to the formation of disulfide bonds induced by excess ROS under oxidative stress, resulting in dimerization of PHD2 and subsequent inactivation [56]. Numerous cell membrane proteins can form disulfide bonds under oxidative conditions, which often impairs their biological functions, such as the regulation of signal transduction and redox homeostasis [57]. For instance, the formation of intermolecular or intramolecular disulfide bonds in pyruvate kinase M2 (PKM2), phosphatase and tensin homolog (PTEN), NF-κB essential modulator (NEMO), and protein tyrosine phosphatases (PTPs) is directly regulated by intracellular ROS levels [58–61]. In the GA-ONFH rats of this study, the level of PHD2 was significantly reduced compared to the untreated control group, demonstrating that elevated ROS levels suppress PHD2 production. Conversely, in rats treated with YGY decoction, the PHD2 level was markedly increased relative to GA-ONFH rats as a result of reduced ROS accumulation within the body. The upregulation of PHD2 expression by the YGY decoction may be attributed to its ability to scavenge excess reactive oxygen species (ROS), thereby blocking ROS-induced intermolecular disulfide bond formation and preserving PHD2 activity. HIF-1α is a key signaling protein that regulates hypoxic responses and plays an important role in bone formation and repair under hypoxic conditions [62, 63]. Under normoxic conditions, the HIF-1α subunit is hydroxylated on two critical proline residues, leading to its recognition by the pVHL and subsequent proteasomal degradation [64]. This process is initiated by specific PHD proteins, namely PHD1, PHD2, and PHD3 [65]. Although the individual subunits of PHD share structural similarity in enzymology in vitro and can all inhibit HIF transcriptional activity, they still exhibit differences [66]. Inhibition of PHD2 through RNA interference can upregulate HIF-1α under normoxic conditions, indicating that PHD2 is a critical factor in regulating HIF-1α in vivo [14, 67]. In both in vivo and in vitro experiments in this study, glucocorticoids were observed to induce an increase in HIF-1α expression, while YGY decoction reduced HIF-1α expression, likely through the ROS/PHD2/HIF-1α signaling pathway. The YGY decoction eliminated excess ROS, and the absence of peroxide interference led to an increase in PHD2 levels, thereby enhancing the ubiquitination process of HIF-1α and reducing its expression. Studies have shown that overexpression of HIF-1α can decrease the expression of the osteogenic marker gene RUNX2, thereby inhibiting osteogenic differentiation, which aligns with our findings [68]. The inhibitory effect of HIF-1α on RUNX2 may be mediated through suppression of TWIST [69]. TWIST, a basic helix-loop-helix transcription factor and a downstream target of HIF-1α, plays a critical role in skeletal tissue development [70]. RUNX2, a key osteogenic regulator, exists in two predominant isoforms (Type 1 [T1] and Type 2 [T2]). Mechanistically, TWIST represses RUNX2 by binding to the E-box sequence within the T1 promoter, thereby suppressing transcription of this isoform and its downstream targets [71]. Under hypoxic conditions, TWIST-mediated inhibition of RUNX2 T1 attenuates RUNX2 expression and downstream signaling in bone marrow mesenchymal stem cells, impairing osteogenic differentiation [72]. In this study, the YGY decoction reduced HIF-1α expression, upregulated the osteogenic marker gene RUNX2, and enhanced osteogenic differentiation. This effect may operate through the HIF-1α/TWIST regulatory axis, wherein ROS scavenging by YGY alleviates hypoxia-driven TWIST activation, consequently rescuing RUNX2-dependent osteogenesis.

**Fig. 9:**
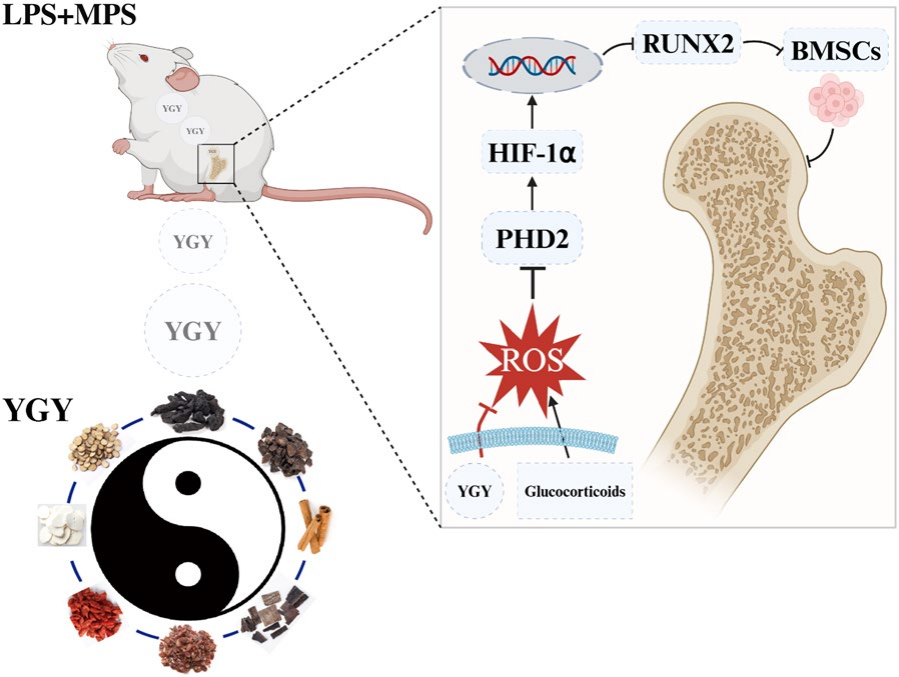
Schematic diagram of the mechanisms by which YGY ameliorates GA-ONFH by regulating the ROS/PHD2/HIF-1α pathway

Based on these scientific findings, as well as network prediction and metabolomics analysis, the ROS/PHD2/HIF-1α signaling pathway may play a crucial role in YGY decoction promoting bone formation in GA-ONFH rats through antioxidative damage. In this study, the expression levels of the osteogenic factor RUNX2 were downregulated in both GA-ONFH rats and DXMS-induced BMSCs, and YGY decoction was able to inhibit this decrease and improve osteonecrosis outcomes both in vivo and in vitro. Additionally, the levels of PHD2 decreased and HIF-1α increased in GA-ONFH rats and DXMS-induced BMSCs, and YGY medicated serum successfully reversed these changes. Finally, we further validated the pathway using gene knockdown methods. These results suggest that YGY decoction effectively counteracts oxidative damage caused by glucocorticoids to achieve therapeutic effects in treating osteonecrosis, which is achieved by modulating the ROS/PHD2/HIF-1α signaling pathway (Fig. 9).

Despite the widespread clinical application of YGY decoction in managing glucocorticoid-induced osteonecrosis with demonstrated therapeutic efficacy, the lack of standardized administration protocols poses challenges for its clinical translation. Key barriers include chemical standardization of the YGY decoction, interspecies dose conversion between animals and humans, and physiological disparities affecting therapeutic outcomes. The effective doses of YGY decoction reported in rodent studies vary significantly across investigations, primarily due to heterogeneity in herbal preparation methods and inter-individual pharmacokinetic variations [20, 73]. Importantly, interspecies differences in ROS metabolism, hypoxia tolerance, and drug absorption may substantially influence YGY decoction’s therapeutic effects in human populations. Notably, murine models exhibit accelerated bone remodeling rates compared to humans, potentially overestimating the osteogenic potency of YGY decoction. While this study confirms YGY’s efficacy in rodent models, several translational hurdles remain unresolved. On one hand, isolation and individual pharmacokinetic profiling of ROS-scavenging constituents are required to establish structure–activity relationships. On the other hand, human metabolic enzymes may differentially process YGY components compared to rodent systems, potentially altering bioavailability and tissue distribution. To bridge these gaps, future translational studies should be prioritized. Firstly, utilize humanized rodent models expressing humanized drug-metabolizing enzymes. Secondly, develop three-dimensional (3D) bone organoids recapitulating human bone microenvironment dynamics. Finally, implement physiologically based pharmacokinetic (PBPK) modeling to predict human dose–response relationships. Building upon this foundation, the translational development of YGY decoction can be better facilitated.

## Conclusion

This study identified 33 bioactive compounds in YGY decoction through comprehensive analysis. Using an integrated approach combining network pharmacology, metabolomics, and experimental validation (both in vitro and in vivo), we demonstrated that YGY-derived compounds mitigate glucocorticoid-induced PHD2 inactivation through ROS scavenging. This mechanism enhances HIF-1α proteasomal degradation, reduces nuclear translocation of HIF-1α, and establishes a positive feedback loop to sustain RUNX2 expression, ultimately promoting osteogenic differentiation. These findings provide mechanistic insights into YGY decoction’s therapeutic potential against glucocorticoid-induced osteonecrosis, bridging traditional medicine with modern molecular pathophysiology. However, large-scale clinical studies are warranted to validate these preclinical findings.

## Supplementary Information

Supplementary Material 1.

## Data Availability

Data used to support the findings of this study are available from the corresponding author upon reasonable request.

## Abbreviations

YGY: Youguiyin
ONFH: Osteonecrosis of the femoral head
SLE: Systemic lupus erythematosus
ROS: Reactive oxygen species
HIF-1α: Hypoxia inducible factor-1α
PHD2: Prolyl hydroxylase 2
p-VHL: Protein- von-Hippel–Lindau
TCM: Traditional Chinese medicine
GA-ONFH: Glucocorticoid-associated osteonecrosis of the femoral head
UPLC-Q-TOF–MS: Ultra-performance liquid chromatography quadrupole time-of-flight mass spectrometry
GO-BP: Gene ontology-biological process
DAVID: Database for annotation, visualization, and integrated discovery
PPI: Protein–protein interaction
LPS: Lipopolysaccharide
MPS: Methylprednisolone
ALN: Alendronate
ELISA: Enzyme-linked immunosorbent assay
WBC: White blood cell count
NEU: Neutrophils
ALT: Alanine aminotransferase
AST: Aspartate aminotransferase
BUN: Blood urea nitrogen
Cr: Creatinine
HE: Hematoxylin–eosin
CVF: Collagen volume fraction
BMSCs: Bone marrow mesenchymal stem cells
SOD: Superoxide dismutase
PTGS: Prostaglandin-endoperoxide synthase
CAT: Catalase
GCLC: Glutamate-cysteine ligase catalytic
GSH: Glutathione
MDA: Malondialdehyde
RUNX2: Runt-related transcription factor 2
