# Supplementary material for: Traditional Chinese medicine Youguiyin decoction ameliorate glucocorticoid-induced osteonecrosis in rat by modulating ROS/PHD2/HIF-1α oxidative stress signaling pathway in bone marrow mesenchymal stem cells
Source: Chin Med. 2025 May 3;20:55. doi: 10.1186/s13020-025-01113-1 (PMC12049805; doi:10.1186/s13020-025-01113-1)
Supplement: Supplementary file 1 — Supplementary Material 1. [file 13020_2025_1113_MOESM1_ESM.doc]

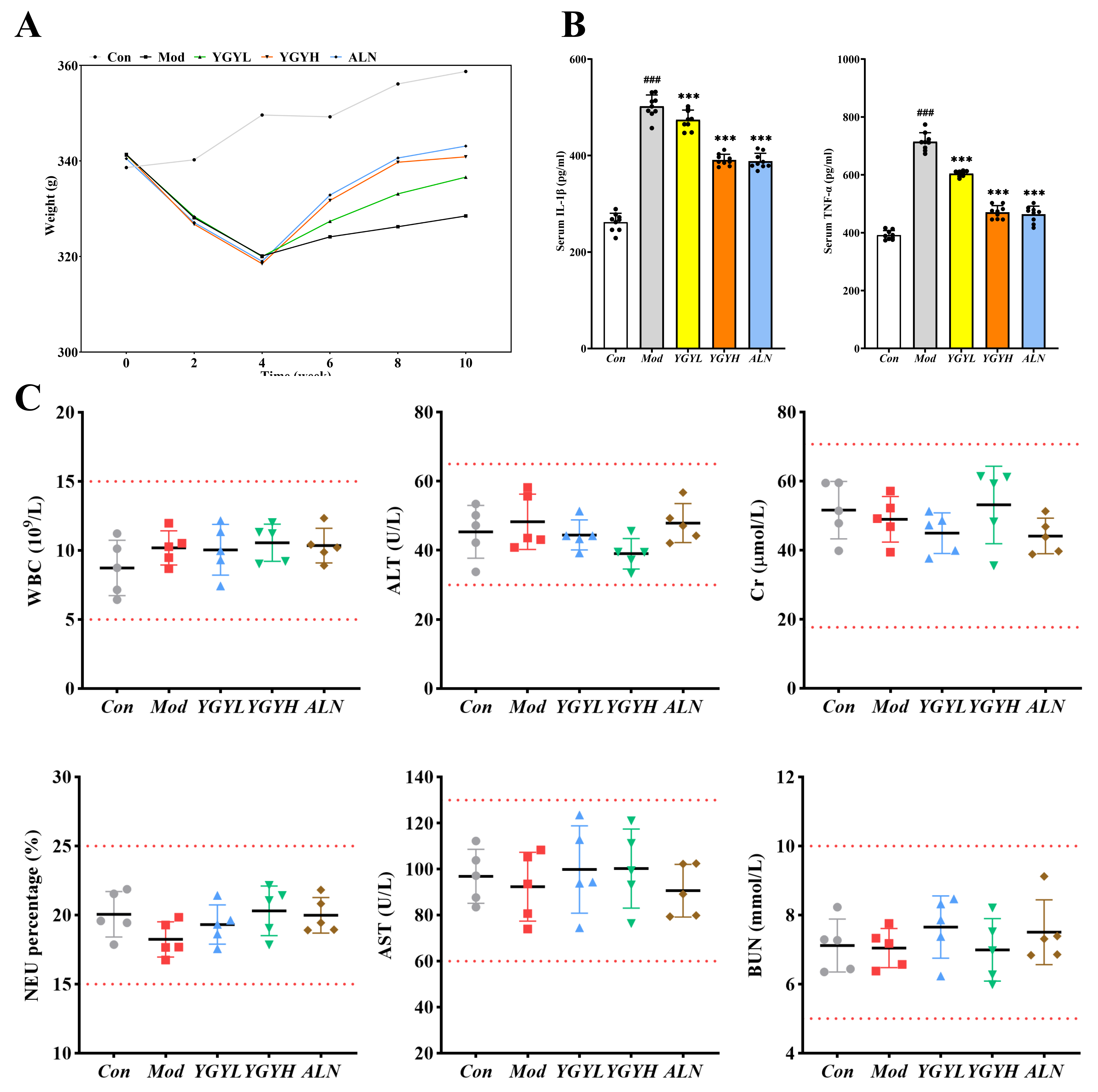


**Figure S1.** Monitoring indices of the animals’ health status. (A) Body weight change curves of rats in each group during the experiment. (B) Levels of inflammatory cytokines IL-1β and TNF-α in serum of rats in each group (n=9 technical replicates). (C) Results of routine blood tests and hepatic and renal function in rats of each group. Statistical analysis: Unpaired t-test. ^###^*P*<0.001 compared with Con group; ****P*<0.001 compared with Mod group.


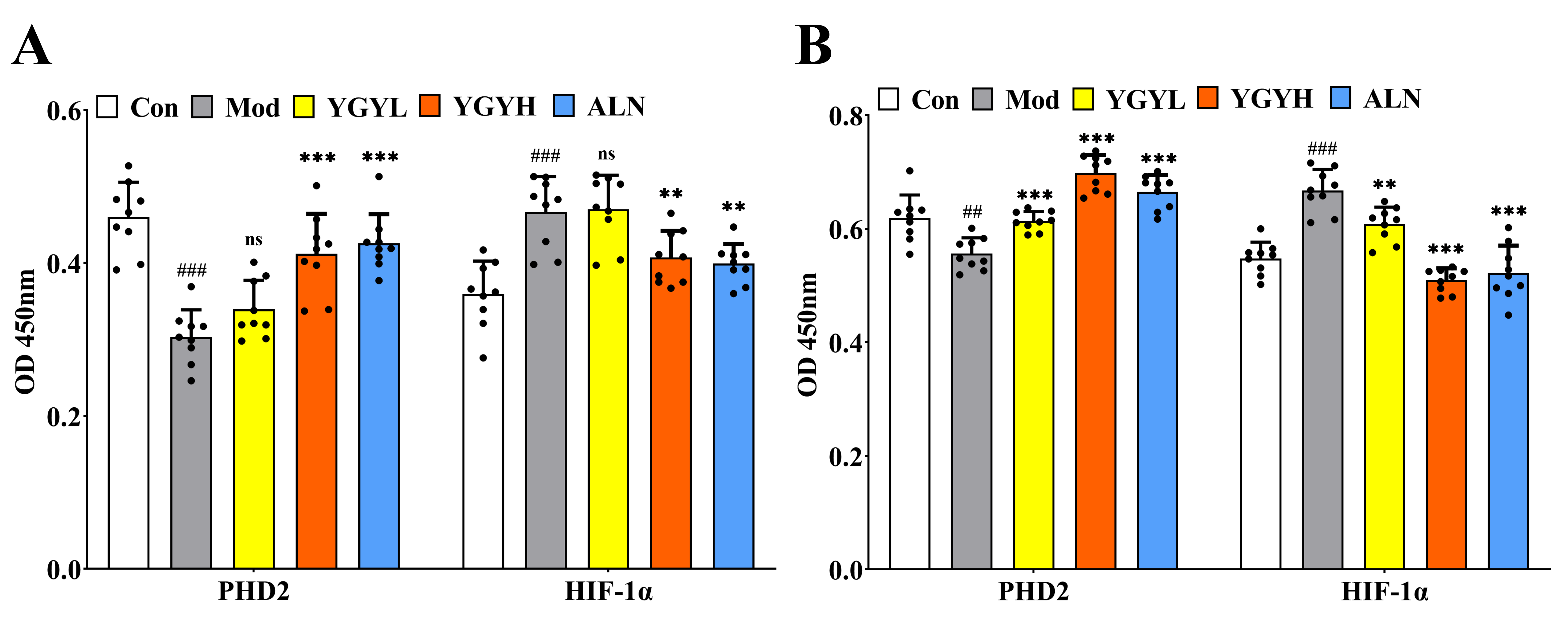


**Figure S2.** PHD2 and HIF-1α levels in the femoral head (A) and serum (B) of rats in each group (n=9 technical replicates). Statistical analysis: Unpaired t-test. ^##^*P*<0.01 and ^###^*P*<0.001 compared with Con group; ^ns^*P*＞0.05,***P*<0.01 and ****P*<0.001 compared with Mod group.


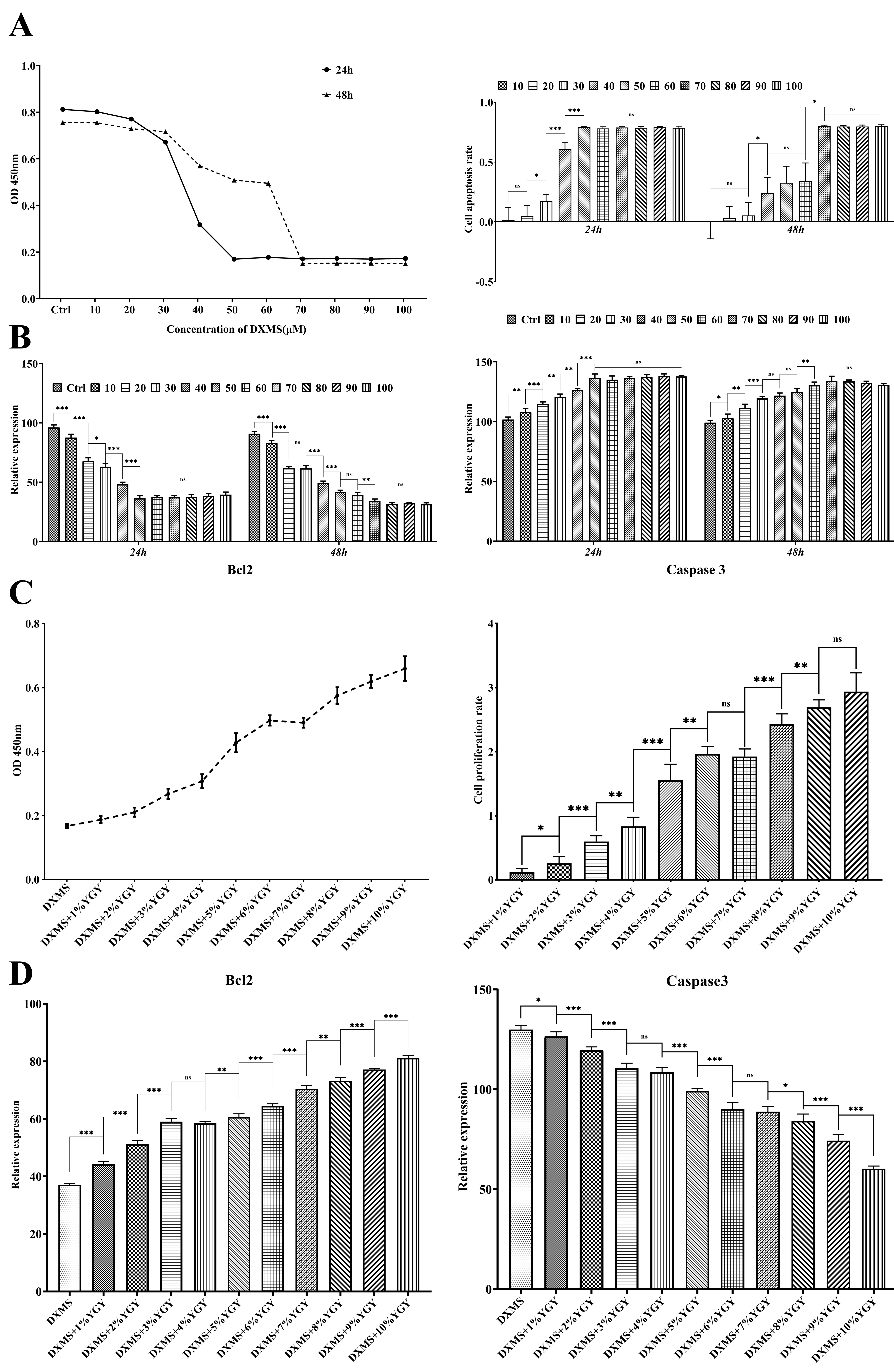


**Figure S3.** The screening process for the concentration of dexamethasone (DXMS) and YGY medicated serum. (A) Results of CCK-8 assay and cell apoptosis rate in BMSCs following dexamethasone (DXMS) intervention at 24h/48h (n=6 technical replicates). (B) The results of PCR detection of the effects of DXMS on the apoptosis genes Bcl2 and Caspase3 in BMSCs (n=6 independent experiments). (C) Results of CCK-8 assay and cell proliferation rate in BMSCs following YGY medicated serum intervention for 24h after pretreatment with 50μM DXMS (n=6 technical replicates). (D) The results of PCR detection showing the effects of different concentrations of YGY medicated serum on the apoptosis genes Bcl2 and Caspase3 in BMSCs induced by 50μM DXMS (n=6 independent experiments). Statistical analysis: Unpaired t-test. ^ns^*P*＞0.05, **P* < 0.05, ***P* < 0.01 and ****P* < 0.001.


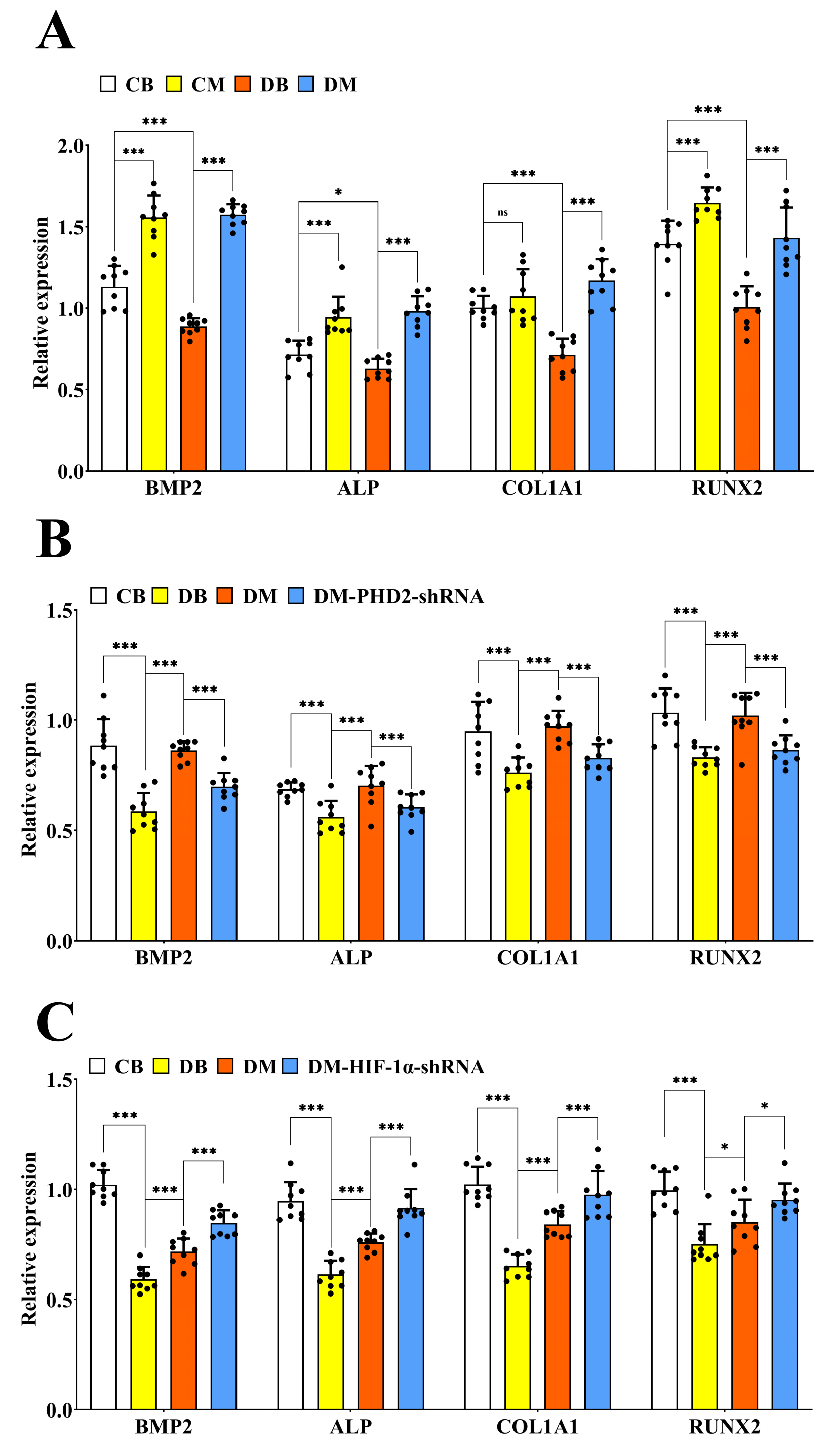


**Figure S4.** PCR results of osteogenic genes in various in vitro experiments. (A) PCR results of osteogenic genes in BMSCs induced by DXMS with intervention of YGY medicated serum. (B) PCR results of osteogenic genes in each group of cells in the in vitro PHD2 gene knockdown experiment. (C) PCR results of osteogenic genes in each group of cells in the in vitro HIF-1α gene knockdown experiment. Statistical analysis: Unpaired t-test (n=9 technical replicates). ^ns^*P*＞0.05, **P* < 0.05, ***P* < 0.01 and ****P* < 0.001.

**Table S1.** YGY decoction associated target genes related to the abundance of components.

| **Numerator ID** | **Component** | **Target gene** | **Abbreviation** |
| --- | --- | --- | --- |
| **MOL006684** | 5,7,3’-trimethoxy - levorotatory - epicatechin | Trypsin-1 | PRSS1 |
| **MOL004319** | Delcosine | Transcription factor p65 | RELA |
| **MOL002752** | Echinacoside | Tumor necrosis factor | TNF |
| **MOL002752** | Echinacoside | Interleukin-6 | IL6 |
| **MOL000415** | Rutin | Caspase-3 | CASP3 |
| **MOL000415** | Rutin | NADPH--cytochrome P450 reductase | POR |
| **MOL000415** | Rutin | Superoxide dismutase [Cu-Zn] | SOD1 |
| **MOL000415** | Rutin | Catalase | CAT |
| **MOL000415** | Rutin | Interleukin-1 beta | IL1B |
| **MOL000785** | Aconine | Interleukin-8 | CXCL8 |
| **MOL001842** | Pinoresinol | Potassium voltage-gated channel subfamily H member 2 | KCNH2 |
| **MOL001842** | Pinoresinol | Sodium channel protein type 5 subunit alpha | SCN5A |
| **MOL001842** | Pinoresinol | Prostaglandin G/H synthase 2 | PTGS2 |
| **MOL001842** | Pinoresinol | Heat shock protein HSP 90 | HSP90 |
| **MOL001842** | Pinoresinol | Nuclear receptor coactivator 2 | NCOA2 |
| **MOL001842** | Pinoresinol | Calmodulin | CALM |
| **MOL001842** | Pinoresinol | Beta-2 adrenergic receptor | ADRB2 |
| **MOL001842** | Pinoresinol | Prostaglandin G/H synthase 1 | PTGS1 |
| **MOL001842** | Pinoresinol | Alpha-1B adrenergic receptor | ADRA1B |
| **MOL001842** | Pinoresinol | Ig gamma-1 chain C region | IGHG1 |
| **MOL001090** | Cinnzeylanol | Coagulation factor VII | F7 |
| **MOL006077** | Hypaconitine | Prostaglandin G/H synthase 1 | PTGS1 |
| **MOL006077** | Hypaconitine | Androgen receptor | AR |
| **MOL006077** | Hypaconitine | Prostaglandin G/H synthase 2 | PTGS2 |
| **MOL005406** | L-Hyoscyamine | Dopamine D1 receptor | DRD1 |
| **MOL005406** | L-Hyoscyamine | Muscarinic acetylcholine receptor M3 | CHRM3 |
| **MOL005406** | L-Hyoscyamine | Muscarinic acetylcholine receptor M1 | CHRM1 |
| **MOL005406** | L-Hyoscyamine | Beta-1 adrenergic receptor | ADRB1 |
| **MOL005406** | L-Hyoscyamine | Muscarinic acetylcholine receptor M5 | CHRM5 |
| **MOL005406** | L-Hyoscyamine | Alpha-2A adrenergic receptor | ADRA2A |
| **MOL005406** | L-Hyoscyamine | Alpha-2C adrenergic receptor | ADRA2C |
| **MOL005406** | L-Hyoscyamine | Muscarinic acetylcholine receptor M4 | CHRM4 |
| **MOL005406** | L-Hyoscyamine | Delta-type opioid receptor | OPRD1 |
| **MOL005406** | L-Hyoscyamine | Sodium-dependent noradrenaline transporter | SLC6A2 |
| **MOL005406** | L-Hyoscyamine | Alpha-1A adrenergic receptor | ADRA1A |
| **MOL005406** | L-Hyoscyamine | Muscarinic acetylcholine receptor M2 | CHRM2 |
| **MOL005406** | L-Hyoscyamine | Alpha-2B adrenergic receptor | ADRA2B |
| **MOL005406** | L-Hyoscyamine | Alpha-1B adrenergic receptor | ADRA1B |
| **MOL005406** | L-Hyoscyamine | Sodium-dependent dopamine transporter | SLC6A3 |
| **MOL005406** | L-Hyoscyamine | Beta-2 adrenergic receptor | ADRB2 |
| **MOL005406** | L-Hyoscyamine | Sodium-dependent serotonin transporter | SLC6A4 |
| **MOL005406** | L-Hyoscyamine | D(2) dopamine receptor | DRD2 |
| **MOL005406** | L-Hyoscyamine | Mu-type opioid receptor | OPRM1 |
| **MOL005406** | L-Hyoscyamine | Gamma-aminobutyric acid receptor subunit alpha-1 | GABRA1 |
| **MOL005406** | L-Hyoscyamine | Histamine H1 receptor | HRH1 |
| **MOL002219** | Atropine | Muscarinic acetylcholine receptor M3 | CHRM3 |
| **MOL002219** | Atropine | Muscarinic acetylcholine receptor M1 | CHRM1 |
| **MOL002219** | Atropine | Muscarinic acetylcholine receptor M5 | CHRM5 |
| **MOL002219** | Atropine | Alpha-2A adrenergic receptor | ADRA2A |
| **MOL002219** | Atropine | Muscarinic acetylcholine receptor M4 | CHRM4 |
| **MOL002219** | Atropine | Delta-type opioid receptor | OPRD1 |
| **MOL002219** | Atropine | Sodium-dependent noradrenaline transporter | SLC6A2 |
| **MOL002219** | Atropine | Alpha-1A adrenergic receptor | ADRA1A |
| **MOL002219** | Atropine | Muscarinic acetylcholine receptor M2 | CHRM2 |
| **MOL002219** | Atropine | Alpha-1B adrenergic receptor | ADRA1B |
| **MOL002219** | Atropine | Sodium-dependent dopamine transporter | SLC6A3 |
| **MOL002219** | Atropine | Beta-2 adrenergic receptor | ADRB2 |
| **MOL002219** | Atropine | Sodium-dependent serotonin transporter | SLC6A4 |
| **MOL002219** | Atropine | D(2) dopamine receptor | DRD2 |
| **MOL002219** | Atropine | Mu-type opioid receptor | OPRM1 |
| **MOL002219** | Atropine | Gamma-aminobutyric acid receptor subunit alpha-1 | GABRA1 |
| **MOL001938** | Secoisolariciresinol | Estrogen receptor | ESR1 |
| **MOL001938** | Secoisolariciresinol | Prostaglandin G/H synthase 2 | PTGS2 |
| **MOL001938** | Secoisolariciresinol | Acetylcholinesterase | ACHE |
| **MOL001938** | Secoisolariciresinol | Beta-2 adrenergic receptor | ADRB2 |
| **MOL001938** | Secoisolariciresinol | Dipeptidyl peptidase IV | DPP4 |
| **MOL001938** | Secoisolariciresinol | Glycogen synthase kinase-3 beta | GSK3B |
| **MOL001938** | Secoisolariciresinol | Heat shock protein HSP 90 | HSP90 |
| **MOL001938** | Secoisolariciresinol | Proto-oncogene serine/threonine-protein kinase Pim-1 | PIM1 |
| **MOL001938** | Secoisolariciresinol | Cyclin-A2 | CCNA2 |
| **MOL001938** | Secoisolariciresinol | Calmodulin | CALM |

**Table S2.** Key nodes and their topological characteristics.

| **Betweenness centrality** | **Degree** | **Name** | **Type** |
| --- | --- | --- | --- |
| **0.00395042** | 2 | RELA | gene |
| **0.00395042** | 2 | TNF | gene |
| **0.00395042** | 2 | IL6 | gene |
| **0.00395042** | 2 | CASP3 | gene |
| **0.00395042** | 2 | SOD1 | gene |
| **0.00395042** | 2 | CAT | gene |
| **0.00395042** | 2 | IL1B | gene |
| **0.00395042** | 2 | CXCL8 | gene |
| **0.03204740** | 4 | PTGS2 | gene |
| **0.00788028** | 2 | NCOA2 | gene |
| **0.04214559** | 5 | ADRB2 | gene |
| **0.02046226** | 3 | PTGS1 | gene |
| **0.01087426** | 2 | AR | gene |
| **0.01203894** | 3 | CHRM2 | gene |
| **0.00468287** | 2 | ADRA2B | gene |
| **0.01203894** | 3 | SLC6A4 | gene |
| **0.01203894** | 3 | DRD2 | gene |
| **0.01203894** | 3 | OPRM1 | gene |
| **0.00468287** | 2 | HRH1 | gene |
| **0.00532609** | 2 | ESR1 | gene |
| **0.00532609** | 2 | DPP4 | gene |
| **0.00532609** | 2 | GSK3B | gene |
| **0.00532609** | 2 | CCNA2 | gene |
| **0.06739911** | 9 | MOL000415 | mol |
| **0.01836145** | 5 | MOL001842 | mol |
| **0.01177008** | 4 | MOL006077 | mol |
| **0.04473238** | 8 | MOL005406 | mol |
| **0.02050157** | 6 | MOL002219 | mol |
| **0.04282729** | 7 | MOL001938 | mol |
| **0.01342690** | 3 | MOL004319 | mol |
| **0.00938103** | 1 | MOL006684 | mol |
| **0.01028193** | 2 | MOL000785 | mol |
| **0.01103940** | 2 | MOL001090 | mol |
| **0.01291932** | 3 | MOL002752 | mol |

**Table S3.** Metabolomic analysis of serum in each group of rats revealed that a total of 10 differential metabolites were significantly upregulated or downregulated after YGY medicated serum intervention.

| **ID** | **Gene** | **General name details** | **Base line (Mod)** | **Base line (YGYH)** |
| --- | --- | --- | --- | --- |
| **P46937*** | YAP | Yes associated protein | 0.776 | 3.571 |
| **Q16665*** | HIF-1α | Hypoxia-inducing factor 1α | 2.399 | 0.801 |
| **Q07812*** | BAX | Bcl-2 associated X protein | 1.457 | 0.330 |
| **P24385*** | Cyclin D1 | G1/S-specific cyclin-D1 | 0.690 | 2.014 |
| **F1M1R0*** | Igkv8-34 | Uncharacterized protein | 0.651 | 1.938 |
| **F1LR92*** | Serpina3m | Serine protease inhibitor A3M | 0.198 | 2.453 |
| **P02680*** | Fgg | Isoform Gamma-A of Fibrinogen gamma chain | 0.767 | 2.600 |
| **D3ZUM5*** | Trim33 | Tripartite motif-containing 33 | 0.539 | 3.379 |
| **D4A4L4*** | Map7d2 | MAP7 domain-containing 2 | 0.141 | 1.618 |
| **Q7TMA5*** | ApoB | Apolipoprotein B-100 | 1.250 | 0.705 |
| **P02651** | Apoa4 | Apolipoprotein A-IV | 0.762 | 1.058 |
| **P02091** | Hbb | Hemoglobin subunit beta-1 | 2.172 | 1.098 |
| **P01015** | Agt | Angiotensinogen | 0.309 | 1.059 |
| **P01048** | Map1 | T-kininogen 1 | 1.875 | 1.076 |
| **Q9R1E9** | Ctgf | Connective tissue growth factor | 0.385 | 0.841 |
| **R9PXW3** | Megf8 | Multiple epidermal growth factor-like domains protein 8 | 0.573 | 1.152 |
| **Q01177** | Plg | Plasminogen | 2.467 | 1.118 |
| **D4A985** | Prdm11 | PR/SET domain 11 | 0.624 | 0.991 |
| **D4A8G5** | Tgf | Transforming growth factor | 0.745 | 1.124 |
| **D4A599** | Dnah17 | Dynein axonemal heavy chain 17 | 0.705 | 1.042 |
| **F1LMP9** | Dab2 | Disabled homolog 2 | 0.744 | 1.077 |
| **D4ACX8** | Dchs1 | Protocadherin-16 | 0.773 | 1.175 |
| **Q8CJ58** | Clrn1 | Clarin-1 | 1.275 | 1.033 |
| **F7FAY5** | LOC360919 | Similar to alpha-fetoprotein | 1.623 | 0.949 |
| **P14630** | Cfi | Complement factor I | 0.747 | 1.056 |
| **P02650** | Apoe | Apolipoprotein E | 1.813 | 0.860 |
| **Q63041** | A1m | Alpha-1-macroglobulin | 1.228 | 0.836 |
| **Q5U2Q8** | Asph | Aspartate-beta-hydroxylase | 1.397 | 1.159 |
| **Q5M878** | Saa4 | Serum amyloid A protein | 1.211 | 0.994 |
| **Q03626** | Mug1 | Murinoglobulin-1 | 2.501 | 1.109 |
| **P50116** | S100a9 | Protein S100-A9 | 0.266 | 1.198 |
| **A2RUW0** | Mroh7 | Maestro heat-like repeat-containing protein family member 7 | 0.076 | 1.024 |
| **Q63207** | F10 | Coagulation factor X | 1.271 | 0.879 |
| **D3ZTE0** | F12 | Coagulation factor XII | 1.210 | 1.044 |
| **P06866** | Hp | Haptoglobin | 1.589 | 0.909 |
| **O35849** | Lcat | Lecithin cholesterol acyltransferase | 0.559 | 1.087 |
| **F1LRE2** | Igfals | Insulin-like growth factor binding protein, acid labile subunit | 0.792 | 1.066 |
| **P15473** | Igfbp3 | Insulin-like growth factor-binding protein 3 | 1.377 | 0.993 |
| **Q5BJM2** | Ccnb1ip1 | Cyclin B1 interacting protein 1 | 0.751 | 1.075 |

**Table S4.** Modeling outcomes across batches

| **Batch** | **Number of modeling** | **Number of osteonecrosis** | **Osteonecrosis rate (%)** |
| --- | --- | --- | --- |
| **Preliminary experiment 1** | 16 | 12 | 75 |
| **Preliminary experiment 2** | 16 | 13 | 81.25 |
| **Formal experiment** | 64 | 48 | 75 |

**Table S5.** Primer sequences for RT-qPCR.

| Primer name | Forward primer (5’-3’) | Reverse primer (5’-3’) |
| --- | --- | --- |
| Bcl2 | TCATGTGTGTGGAGAGCGTC | AGTTCCACAAAGGCATCCCAG |
| Caspase3 | GAGCTTGGAACGCGAAGAAA | TTGCGAGCTGACATTCCAGT |
| BMP2 | GGACCCGCTGTCTTCTAGTG | TTCCTCGATGGCTTCTTCGT |
| ALP | TTGGTGGAAACAGCCATCTGA | GCGTTGGTGTTGTACGTCTTG |
| COL1A1 | ATGTTCAGCTTTGTGGACCTC | CACCGACAGCACCATCGTTA |
| β-actin | AGCCTTCCTTCCTGGGTATGG | AAGGGTGTAAAACGCAGCTC |

**Table S6.** Antibodies list.

| **Name** | **Citation** | **Supplier** | **Cat no.** | **Dilution ratio** |
| --- | --- | --- | --- | --- |
| **PHD2** | WB | Proteintech | 19886-1-AP | 1:5000 |
| **HIF-1α** | WB | Proteintech | 20960-1-AP | 1:2000 |
| **Runx2** | WB | Proteintech | 20700-1-AP | 1:1000 |
| **β-actin** | WB | Proteintech | 81115-1-RR | 1:10000 |
